# Metabolic features that select for Bathyarchaeia in modern ferruginous lacustrine subsurface sediments

**DOI:** 10.1093/ismeco/ycae112

**Published:** 2024-09-14

**Authors:** Fátima Ruiz-Blas, Alexander Bartholomäus, Sizhong Yang, Dirk Wagner, Cynthia Henny, James M Russell, Jens Kallmeyer, Aurèle Vuillemin

**Affiliations:** GFZ German Research Centre for Geosciences, Section Geomicrobiology, Telegrafenberg, 14473 Potsdam, Germany; GFZ German Research Centre for Geosciences, Section Geomicrobiology, Telegrafenberg, 14473 Potsdam, Germany; GFZ German Research Centre for Geosciences, Section Geomicrobiology, Telegrafenberg, 14473 Potsdam, Germany; GFZ German Research Centre for Geosciences, Section Geomicrobiology, Telegrafenberg, 14473 Potsdam, Germany; University of Potsdam, Institute of Geosciences, Karl-Liebknecht-Str. 24-25, Potsdam 14476, Germany; Research Center for Limnology and Water Resources, National Research and Innovation Agency (BRIN), Jl. Raya Bogor Km. 46 Cibinong, Bogor 16911, West Java, Republic of Indonesia; Department of Earth, Environmental, and Planetary Sciences, Brown University, 324 Brook Street, Providence, RI 02912, United States; GFZ German Research Centre for Geosciences, Section Geomicrobiology, Telegrafenberg, 14473 Potsdam, Germany; GFZ German Research Centre for Geosciences, Section Geomicrobiology, Telegrafenberg, 14473 Potsdam, Germany

**Keywords:** ferruginous sediment, Bathyarchaeia, fermentation, remineralization, methanogenesis, (homo)acetogenesis, cryptic sulfur cycling, metagenome-assembled genomes, Lake Towuti

## Abstract

Ferruginous conditions prevailed through Earth’s early oceans history, yet our understanding of biogeochemical cycles in anoxic iron-rich, sulfate-poor sediments remains elusive in terms of redox processes and organic matter remineralization. Using comprehensive geochemistry, cell counts, and metagenomic data, we investigated the taxonomic and functional distribution of the microbial subsurface biosphere in Lake Towuti, a stratified ferruginous analogue. Below the zone in which pore water becomes depleted in electron acceptors, cell densities exponentially decreased while microbial assemblages shifted from iron- and sulfate-reducing bacterial populations to fermentative anaerobes and methanogens, mostly selecting Bathyarchaeia below the sulfate reduction zone. Bathyarchaeia encode metabolic machinery to cycle and assimilate polysulfides via sulfhydrogenase, sulfide dehydrogenase, and heterodisulfide reductase, using dissimilatory sulfite reductase subunit E and rubredoxin as carriers. Their metagenome-assembled genomes showed that carbon fixation could proceed through the complete methyl-branch Wood-Ljungdahl pathway, conducting (homo)acetogenesis in the absence of methyl coenzyme M reductase. Further, their partial carbonyl-branch, assumed to act in tetrahydrofolate interconversions of C1 and C2 compounds, could support close interactions with methylotrophic methanogens in the fermentation zone. Thus, Bathyarchaeia appeared capable of coupling sulfur-redox reactions with fermentative processes, using electron bifurcation in a redox-conserving (homo)acetogenic Wood-Ljungdahl pathway, and revealing geochemical ferruginous conditions at the transition between the sulfate reduction and fermentation zone as their preferential niche.

## Introduction

Ferruginous (iron-rich, sulfate-poor) conditions dominated much of Earth’s early oceans history [1] before sulfate became available and abundant [2]. While the geochemistry of past marine environments has been studied extensively, metabolic activities of a microbial biosphere slowly buried in the subsurface remain elusive in sedimentary records [3, 4]. Recognizing the major biogeochemical transitions, such as oceanic bottom water oxygenation [5] and the rise of seawater sulfate [6] during the Proterozoic Eon, requires empirical constraints on microbial processes in terms of redox changes and organic matter (OM) mineralization of sediment substrates during early diagenesis [7]. Although ferruginous geochemical conditions are common in sediment pore water below the depth of sulfate depletion [8], modern sedimentary environments in which to study microbial communities and metabolic processes analogous to those that operated in Proterozoic oceans enriched in iron oxides and devoid of sulfate, are scarce [9, 10]. Moreover, modern sediments contain substantially more primary OM and their iron sources are rather of detrital than hydrothermal origin, usually orienting the system towards methanogenesis as the main remineralization process [11].

**Figure 1:**
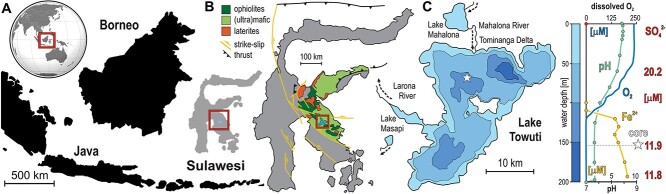
**Site description of Lake Towuti.** (**A**) World map displaying the location of Sulawesi Island (square), with close-up on the Indonesia archipelago and location of the Malili Lake system (square). (**B**) Map of Sulawesi illustrating the geological context of the Malili Lake system (square). (**C**) Bathymetric map of Lake Towuti with position of the coring site (star) corresponding to 156 m water depth; oxygen, iron, pH profiles and sulfate concentrations of Lake Towuti’s water column [15]. The figure is modified from [17].

Ferruginous sediments are deposited in the Malili Lakes, a chain of five interconnected tectonic lakes hosted in ultramafic rocks on Sulawesi Island, Indonesia (Fig. 1A). Weathering of lateritic soils in the catchment (Fig. 1B) supplies considerable amounts of iron (oxyhydr)oxides [12], but very little sulfate to the lakes [13]. These iron oxides scavenge phosphorus in the water column and limit its productivity [14]. Lake Towuti (2.5° S, 121.3° E), the largest of the Malili Lakes, is currently stratified, displaying oligotrophic conditions with persistent ferruginous anoxia in its bottom waters (Fig. 1C). A substantial part of the sinking OM is remineralized through microbial reduction of ferrihydrite [15], and potentially methanogenesis [11], suggesting analogies to depositional processes in early ferruginous ecosystems [9]. Previous geomicrobiological investigations showed that, despite extremely low sulfate concentrations (<20 μM), biogeochemical processes related to sulfur, iron and methane co-occur in sediments [10, 16], which thereby may provide clues to early diagenesis by selective benthic microbial communities.

Using metagenomic data, we investigate Lake Towuti’s subsurface biosphere in terms of environmental selection, and identify metabolic traits that reflect specific adaptation to anoxic ferruginous conditions. We report bulk sediment, methane and pore water geochemical data along a sediment core spanning ~3000 years of sediment history [18]. Using sulfate reduction rate (SRR), cell count, 16S rRNA gene and metagenomic sequencing data, we characterize the abundance, taxonomic distribution, and metabolic functions of microbial communities, and trace their assembly during burial in ferruginous deposits. By adding taxonomy of functional marker genes and analysis of metagenomes-assembled genomes (MAGs), we determine which metabolic features among the main microbial clades relate to processes of metal and sulfate reduction, OM remineralization, and methanogenesis. Finally, we detail the metabolic machinery that selects Bathyarchaeia in sediments and consider its relevance to a primeval biosphere in Earth’s ferruginous oceans.

## Materials and methods

### Field sampling and sediment aliquoting

Field sampling took place in October 2013, November 2014, and June 2015, allowing the study of geochemical conditions in the water column and collection of sediment gravity cores. Oxygen concentration profiles of the water column were measured using a SBE-19 conductivity-temperature-depth probe (Sea-Bird Electronics, Bellevue, Washington, USA) [15]. Fe^2+^ concentrations (Fig. 1C) were obtained from water samples collected using 5 l Niskin bottles (General Oceanics, Miami, USA) [15]. Sediments of the upper 55 cm below lake floor (cmblf) were retrieved from 156 m water depth (Fig. 1C) via gravity coring [16]. In the field, gravity cores were transferred into an anaerobic glove bag and sediments subsampled for methane (2 cm^3^), SRR (3 × 5 cm^3^), pore water geochemistry (50 cm^3^), cell count (2 cm^3^), and DNA extraction (full core). Samples for methane were taken with a cutoff syringe immediately after core retrieval, and stored in crimp vials filled with saturated NaCl solution without headspace. Sample resolution was 1 cm for the upper 5 cmblf, 2 cm for 5 to 20 cmblf, and 5 cm below. For DNA samples, a separate gravity core was aliquoted by placing bulk sediment into heat-sealed gas-tight aluminum-foil bags flushed with nitrogen [16] and stored at room temperature (25°C), close to that of lake bottom waters (28°C), until analysis. Sequencing of 16S rRNA gene libraries performed on biological replicates in 2015, 2019, and 2022 yielded identical results [10,19], ensuring that long-term storage at room temperature did not alter the microbial composition of sediment samples. Detailed procedures for downstream geochemical analyses were previously published [3,16,17].

### Bulk sediment, pore water geochemistry, and methane concentrations

Total organic carbon (TOC), total nitrogen (TN), total carbon (TC), and total sulfur (TS) concentrations were measured on freeze-dried bulk sediment. Pore water was extracted in the glove bag by transferring sediments into 50 ml Falcon tubes, using Rhizon Pore Water Samplers (Rhizosphere research products, Wageningen, Netherlands). The pore water was sterile-filtered, transferred into a plastic cryovial with screw cap (Cryo Vials, Carl Roth, Karlsruhe, Germany) and stored at 4°C until analysis.

Pore water Ca^2+^, Mg^2+^, Cl^−^, SO_4_^2−^, and NH_4_^+^ concentrations were analyzed in triplicates using non-suppressed (cations) and suppressed (anions) ion chromatography [16]. Detection and quantification limits of the method are 8.3 and 38.5 μM for Ca^2+^; 9.6 and 44.6 μM for Mg^2+^; 5.7 and 16.2 μM for Cl^−^; 2.0 and 8.4 μM for SO_4_^2−^; and 11.3 and 67.6 μM for NH_4_^+^. Dissolved Fe^2+^ concentrations were measured in the field, transferring 1 ml of pore water aliquots to 1.5 ml single-use cells (Rotilabo, Carl Roth) stabilized with 100 μl of Ferrozine Iron Reagent (Sigma-Aldrich, Taufkirchen, Germany). The absorbance of the colored solution was measured at 562 nm [20] with a DR 3900 spectrophotometer (Hach, Düsseldorf, Germany). For methane concentrations, we introduced 3 ml of helium in the crimp vials as headspace 24 h prior to analysis, and injected 200 μl of headspace into a Thermo Finnigan Trace gas chromatograph (Thermo Fisher Scientific), as published [11].

### Total cell counts and sulfate reduction rates

For total cell counts, 2 cm^3^ of sediment were fixed in 8 ml formalin solution (final concentration 2%). From this slurry, we mixed 50 μl with 50 μl detergent mix (i.e. 36.8 g l^−1^ Na_2_ EDTA × 2 H_2_O, 22.3 g l^−1^ Na-pyrophosphate × 10 H_2_O, 5 ml TWEEN 80), 50 μl methanol and 350 μl ultrapure H_2_O, and processed triplicates [21]. To dissolve fine mineral particles, 50 μl of this solution were mixed with 5 μl of 1% hydrofluoric acid [22]. The solution was filtered onto black 0.2 μm polycarbonate Cyclopore membrane filters (Whatman International Ltd, Maidstone, United Kingdom), cells stained with SYBR Green I (Molecular Probes, Eugene, Oregon, USA), and counted by epifluorescence microscopy (Leica DM2000, Wetzlar, Germany), as published [16].

SRR were determined by sediment incubation with radioactive ^35^SO_4_^2−^ using sterile glass barrels (ca. 5 cm^3^) processed in triplicates [11]. The microbially-reduced inorganic sulfur species were separated using a cold chromium distillation [23] and radioactivity in the extracts quantified via scintillation counting using Ultima Gold Scintillation Cocktail (Perkin Elmer, Waltham, Massachusetts, USA) and a TriCarb 2500 TR liquid scintillation counter (Packard Instruments, Fallbrook, California, USA).

### DNA extraction, 16S rRNA gene library preparation and analysis

Total DNA was extracted from 1 g of sediment using the GeneMATRIX Environmental DNA & RNA Purification Kit (EURx, Gdánsk, Poland), following the manufacturer’s instructions. PCR amplification was performed as published [19], using unique barcoded combinations of the universal primer pair 515F (5′-GTG TGY CAG CMG CCG CGG TAA-3′) with 806R (5′- CCG GAC TAC NVG GGT WTC TAA T-3′). PCR products were cleaned with the HighPrep PCR Clean-up system (MAGBIO, Lausanne, Switzerland) and sent to Novogene (www.novogene.com) for 16S rRNA gene sequencing (2 × 250 bps) on an Illumina NovaSeq platform (Illumina, San Diego, California, USA). Read demultiplexing was performed using Cutadapt v. 3.5 [24] with the following parameters: -e 0.2 -q 15,15 -m 150 --discard-untrimmed. The amplicon sequence variants (ASVs) were generated using trimmed reads and the DADA2 package v. 1.20 [25] with R v. 4.1, applying the pooled approach with the following parameters: truncLen = c(220 180), maxN = 0, rm.phix = TRUE, minLen = 160. Taxonomic assignment was done using DADA2 against the SILVA 16S rRNA SSU database release 138 [26]. ASVs representing chloroplasts, mitochondria and singletons were removed. The partial 16S rRNA gene sequences were aligned using SINA online v. 1.2.11 [27] and inserted into a Maximum Likelihood RAxML phylogenetic tree on ARB [28], using the maximum parsimony algorithm with the bacterial and archaeal filters, and selecting the best tree among 100 replicates. We perform further phylogenetic analysis at deeper taxonomic level to support interpretations of 16S rRNA genes in terms of functional guilds. ASVs corresponding to putative methanogens, methanotrophs, sulfate-reducing bacteria (SRB), Chloroflexota, and Bathyarchaeia were plotted into five distinct phylogenetic trees (Supplementary Figs. S1–S5), and the related 16S rRNA genes and metagenomic reads plotted as bar charts to assess the relative abundance of the corresponding functional guilds (Supplementary Fig. S6).

A canonical correspondence analysis (CCA) was performed with 15 explanatory variables and the 1000 most abundant ASVs, using Past v. 4.03 [29]. Significance of canonical axes was tested via permutation (N = 999). CCA results were cross-checked via two non-metric multidimensional scaling (NMDS) analyses based on 4559 ASVs and 138 243 Open Reading Frames (ORFs) (Supplementary Fig. S7).

### Metagenome processing and extraction of functional genes as markers

Eight metagenomes were generated from DNA extracts (at 0–1, 2–4, 6–8, 10–12, 14–16, 20–25, 30–35, and 40–45 cmblf), using the Nextera XT DNA Library Preparation kit (Illumina). Sequencing was performed on a NovaSeq 6000 Illumina platform at CeGaT GmbH (Tübingen, Germany), aiming for 50 million read pairs (2 × 150 bps) for each sample. Library demultiplexing was performed with bcl2fastq2 v. 2.20. Adapters were trimmed with Skewer v. 0.2.2 [30], and FASTQ files quality-checked using FastQC v. 0.11.5. Quality-controlled reads were mapped to the SILVA 16S rRNA SSU database release 138 [26] using Bowtie2. Metagenomic reads were further processed for quality control, de novo assembly of contigs, gene annotation, binning into MAGs and taxonomic annotation, using ATLAS v. 2.1.0 [31]. Metagenomes were initially processed separately, then combined to improve the completeness of MAGs. Briefly, the integrated ATLAS pipeline uses quality-controlled reads from BBTools to successively execute: metaSPAdes v. 3.11.1 for contig assembly; Prodigal v. 2.6.3 for ORFs extraction; eggNOG-mapper v. 2.1 for functional annotation; MetaBAT v. 2.1.5 with MaxBin v. 2.2.7 and DAS Tool v. 1.1.6 for binning into MAGs; and CheckM v.1.1.10 to determine the level of MAG completeness and contamination. All aforementioned software, scripts and databases are integrated in the ATLAS pipeline (Supplementary Methods). Taxonomic assignments of the MAGs are performed against the GTDB Genome Taxonomy Database v. 2.1.1. In addition, we extracted 16S rRNA gene sequences that could be assembled in our MAGs and plotted them in a phylogenetic tree (Supplementary Fig. S8), as described above. Taxonomic assignment of 17 MAGs to Bathyarchaeia orders was further confirmed via phylogenetic analysis of 16 concatenated ribosomal protein sequences (Supplementary Fig. S9) against the 286 representative MAGs of Bathyarchaeia from the GTDB database, according to published scripts [32]. An anvi’o pangenome analysis [33] was then carried out on these 17 MAGs with 22 GTDB representative MAGs of Bathyarchaeia (Supplementary Fig. S10).

Taxonomic identifications integrated with functional annotations were performed on all ORFs extracted from contigs assembled from separate metagenomes, as well as all ORFs extracted from MAGs, using DIAMOND protein aligner v. 0.9.24. [34]. BLASTp searches were run against an aggregated genome database of 37.8 million predicted proteins, including the SEED and NCBI RefSeq databases, and taxonomy of the best hit assigned to the corresponding ORF, as published [35]. We were thus able to attribute the microbial provenance of all ORFs extracted from contigs to high taxonomic levels and exert comparison with the genetic content of MAGs assembled from combined metagenomes.

In both cases, we extracted ORFs encoding proteins involved in dissimilatory sulfate to polysulfide reduction (*sat*, *apr*, *dsr*, *asr*, *phs*/*psr*, *hyd*), and sulfide dehydrogenase (*sud*), also known as bifurcating ferredoxin: NADP oxidoreductase (*nfn*). Concerning the Wood-Ljungdahl (W-L) pathway [36], we extracted genetic information on all ORFs encoding proteins related to the carbonyl-branch (*fdh*, *fts*, *mthfc/mthfd*, *mthfr*, *mthmt*) and methyl-branch (*fmd*, *ftr*, *mch*, *mtd*, *mer*) and their common final steps (*codh*, *cdha*). For production/consumption of the end products acetate and methane, we looked for phosphotransacetylase (*pta*), acetate kinase (*akn*), acetyl-CoA synthetase (*acs*) and acyl-CoA synthetase short chain (*acss2*), and coenzyme M methyltransferases (*mta*, *mtb*, *mts*, *mtr*) with methyl-coenzyme M reductase (*mcr*), respectively. Iron-related genes were identified using the FeGenie bioinformatic tool [37]. To assess the use of molecular hydrogen in energy-conserving proton and sodium pumps [38, 39], we extracted ORFs encoding specific hydrogenases (*frh*, *mvh*, *hdr*), redox enzymes functioning as cofactors of the electron transfer chain (*trx*, *rbx*, *grx*, *ald-fdx*, *2-oxo-fdx*, *pyr-fdx*), and flavin-based electron bifurcation (*Ech*, *Rnf*, *Nuo*) complexes [40]. Finally, we looked for ribulose-1,5-diphosphate carboxylase-oxygenase (*RuBisCO*) with the formaldehyde-activating enzyme (*fae*) and thymidine/AMP phosphorylase (*deoA*) to assess putative *RuBisCO*-mediated pathways in archaea [41, 42]. The full list of enzymes and gene abbreviations is available as supplement (Supplementary Table S1).

## Results

### Substrate depletion with sediment depth: geochemistry, cell densities, and SRR

**Figure 2:**
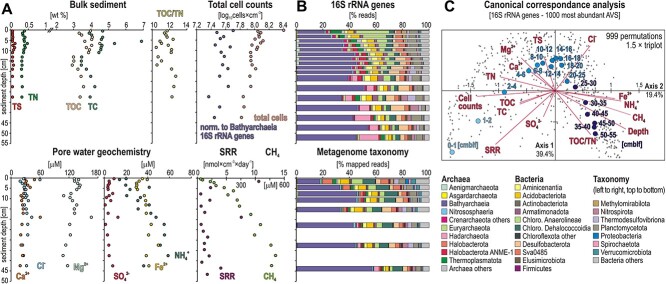
**Depth profiles for sediment and pore water geochemistry, taxonomy of microbial populations and** **canonical correspondance analysis**. (**A**) Total sulfur (TS), total nitrogen (TN), total organic carbon (TOC), and total carbon (TC) in bulk sediment (**top**); TOC/TN ratios; total cell counts and total cell counts normalized to 16S rRNA gene abundances of Bathyarchaeia (**top**); pore water concentrations in calcium (Ca^2+^), chlorine (Cl^−^), magnesium (Mg^2+^), sulfate (SO_4_^2−^), ammonia (NH_4_^+^), and ferrous iron (Fe^2+^); sulfate reduction rates (SRR) and methane (CH_4_) concentrations (**bottom**). (**B**) Relative abundances of 16S rRNA genes (**top**) and metagenomic read taxonomic assignments (**bottom**). (**C**) Canonical correspondence analysis (CCA) based on the 15 environmental variables listed above and the 1000 most abundant amplicon sequence variants (ASVs). The distribution of ASVs (dots) plots according to the sediment depth of each successive sample (open circles) in cm below lake floor (cmblf). Environmental variables (triplot) that significantly influence sample distribution with sediment depth are SRR, cell counts, Fe^2+^, NH_4_^+^, and CH_4_ concentrations.

The depth of the oxycline [15, 16] and redox conditions at the sediment–water interface (SWI) result in variable burial of ferric iron and OM over time [3], determining sediment substrates available to microbial communities during burial. Despite oligotrophic conditions in the water column, the sediment organic content is relatively high. TS, TN, TOC, and TC concentrations fluctuate around 0.1, 0.5, 3.5, and 4.5 [weight %], respectively (Fig. 2A). A siderite (FeCO_3_) layer (7 wt % TC) occurs around 8 cmblf. TOC/TN ratio values fluctuate between 11.7 and 12.7, reflecting the admixture of terrestrial and algal OM sources. Continuous anaerobic degradation of labile OM occurs in the sediment as shown by NH_4_^+^ concentrations increasing from 20 μM at the SWI to 60 μM at 40 cmblf. Because NO_3_^−^ concentrations were below detection (<4.1 μM), we infer microbial breakdown of OM as the main source of pore water NH_4_^+^. Methane concentrations increase constantly with sediment depth from 0 at the SWI to ca. 550 μM in the bottom core (Fig. 2A).

In the (near) absence of O_2_, NO_3_^−^ and SO_4_^2−^ in ferruginous bottom waters (Fig. 1C), electron acceptors in the pore water are depleted at only few cmblf. The reduction of reactive ferric phases (e.g. ferrihydrite) leads to an increase in dissolved Fe^2+^ concentrations from 17 μM at the SWI to a maximum of 49 μM at 32.5 cmblf, whereas other detrital iron phases (e.g. goethite) are poorly reactive towards microbial reduction [11]. Lake Towuti’s bottom waters exhibit very low SO_4_^2−^ concentrations (~12 μM) that further decrease in pore water from 9 μM at the SWI to ~5 μM (detection limit: 2.0 μM) at 14 cmblf, only fluctuating in the remainder of the core (Figs. 1C and2A). Pore water Ca^2+^, Cl^−^, and Mg^2+^ concentrations are respectively ca. 30, 30 and 130 μM, indicating stable alkaline freshwater conditions (Fig. 2A).

The density of microbial populations and SRR are directly impacted by the depletion of pore water electron acceptors and labile OM [18]. Total cell counts (Fig. 2A) display a logarithmic decrease down to 45 cmblf (log_10_ = 8.4 to 7.8). In comparison, cell counts normalized to Bathyarchaeia 16S rRNA genes remain quite constant (log_10_ = 7.7 to 7.4). Despite very low pore water SO_4_^2−^ concentrations in sediments, radiotracer incubation experiments revealed measurable SRR in the upper 50 cmblf. The SRR were 10 nmol × cm^−3^ day^−1^ at the SWI, decreasing to ca. 1 nmol × cm^−3^ day^−1^ below (Fig. 2A). Our ^35^SO_4_^2−^ radiotracer experiments clearly indicate that microbial sulfate reduction, and potentially cycling of sulfur intermediates [43], is taking place.

Overall, the profiles of pore water geochemistry, SRR and methane concentrations indicate a metabolic transition from sulfate reduction to methanogenesis around 5–10 cmblf (Fig. 2B), with a concomitant decrease in cell densities in the fermentative zone [44]. The depletion of reactive ferric minerals and organic substrates over ~3000 years of sedimentation was expected to exert subsequent control on the distribution of microbial clades [45–48].

### Subsurface biosphere assembly: taxonomic diversity of 16S rRNA genes and metagenomes

Taxonomic assignments of 16S rRNA gene amplicons from 18 samples resulted in 462 588 processed sequences and 4559 different ASVs, of which 1594 ASVs (~35%) were identified as Archaea and 2965 ASVs (~65%) as Bacteria (Fig. 2B). Bar charts of 16S rRNA genes and metagenomic reads [relative read %] indicate that 5 to 15% of microbial clades (Desulfobacterota, Thermodesulfovibrionia) are closely affiliated with putative SRB, whereas concomitant functional guilds related to putative methanotrophs (ANME-1) and methanogens (Euryarchaeota, Halobacterota) amount to <10% of the total community (Supplementary Fig. S6). The partial 16S rRNA gene phylogenetic trees confirmed that many ASVs are taxonomically close to putative SRB, methanotrophs, and methanogens (Supplementary Figs. S1–S3). In detail, the functional guild corresponding to SRB is composed of the following ASVs: 237 Desulfobacterota, 6 Firmicutes and 62 Thermodesulfovibrionia (phylum Nitrospirota). The methanogen functional guild includes 33 Methanomassiliicoccales (phylum Thermoplasmatota), 34 Methanofastidiosales and Methanobacteriales (phylum Euryarchaeota), 6 Methanomethyliales (phylum Crenarchaeota), 20 diverse Halobacterota, while 19 ANME-1 constituted the functional guild of methanotrophs. Finally, 631 ASVs are assigned to clades of Chloroflexota (Supplementary Fig. S4) and 965 ASVs to different groups of Bathyarchaeia (Supplementary Fig. S5). The relative abundance of these functional guilds (SRB, methanogens, methanotrophs) decreases to around 5% with depth (Supplementary Fig. S6). In comparison, Bathyarchaeia progressively prevail, increasing from ~20% at the SWI to >50% of the total community in deeper sediments. Hadarchaeota also become more abundant (>15%), whereas Chloroflexota drop from 30% at the SWI to <10% in lowermost sediments (Fig. 2B). Despite some discrepancies, the taxonomy of metagenomic reads is consistent with 16S rRNA genes, e.g. Bathyarchaeia are more represented in metagenomic reads and increase steadily with depth, Altiarchaeota account for 5% of the population in the upper 5 cmblf, Hadarchaeota are less abundant below 20 cmblf, and Chloroflexota remain the most abundant bacteria throughout depth (Fig. 2B).

The CCA analysis correlates cell counts, SRR, Fe^2+^, NH_4_^+^ and CH_4_ concentrations with the plotted distribution of ASVs (Fig. 2C) as a function of sediment depth. The triplot and ASV distribution confirm that the depletion of pore water electron acceptors and gradual OM degradation induce a decrease in cell number and SRR while pore water concentrations of metabolic end products keep increasing. Thus, the axes 1 (39.4%) and 2 (19.4%) can be interpreted in terms of sediment depth and geochemistry, the U-turn along the axis 2 suggesting a transition from sulfate reduction to fermentation around 10–15 cmbf. The selective assembly of a subsurface biosphere in terms of taxonomic (4559 ASVs) and functional (138 243 ORFs) composition is reflected in the two NMDS plots, displaying a depth-dependent distribution of samples (Supplementary Fig. S7).

De novo assembly of the separate metagenomes produced, on average per library, 75 517 938 reads assembled into 35 772 contigs, with 123 049 ORFs extracted (Supplementary Table S2). Their combined de novo assembly included 603 920 334 reads assembled into 499 820 contigs, with 1 145 704 ORFs extracted, resulting in genomic binning of 70 MAGs (Fig. 5A), of which 51 were considered good-quality (>70% completeness; <10% contamination), for a total of 93 247 predicted ORFs. Taxonomic assignments of these 70 MAGs identified 31 Archaea and 39 Bacteria (Supplementary Tables S3 and S4), namely: 17 Bathyarchaeia (B24, B26-1, 40CM-2-53-6, TCS64, RBG-16-48-13) [49], 1 Nitrososphaeria, 2 Methylarchaeales [50], 2 Brockarchaeota [51], 1 *Candidatus* Verstraetearchaeota [52], 1 Euryarchaeota, 1 Hadarchaeota, 3 Halobacterota, 1 Nanoarchaeota, and 3 Thermoplasmatota; and 3 Aminicenantia, 2 Acidobacteriota, 1 Actinobacteriota, 1 Bacteroidota, 12 Chloroflexota, 6 Desulfobacterota, 2 Methylomirabilota, 6 Nitrospirota (5 Thermodesulfovibrionia), 3 Planctomycetota, 1 Zixibacteria, and 1 bacterial clade TA06.

### Functional diversity with sediment depth: selective metabolic traits in metagenomes

To assess the evolution of metabolic potential as a function of sediment depth, we plotted the percentage of predicted ORFs assigned to functional genes for each separate metagenome [% ORFs] along with their taxonomic assignments [relative %] at the phylum/class level. We acknowledge that the absence of specific marker genes may result from limited sequencing depths.

**Figure 3:**
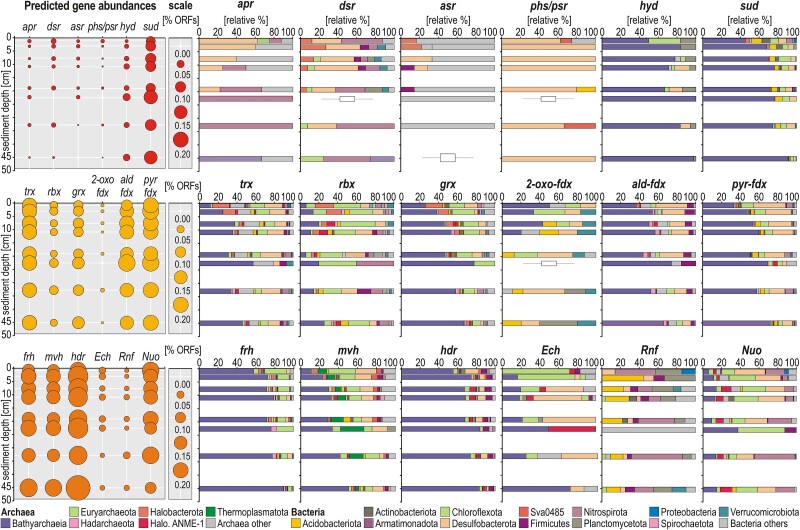
**Relative abundances of functional marker genes related to sulfur cycling, redox cofactors and the use of molecular hydrogen and their corresponding taxonomic assignments.** Relative abundances (**left**) and taxonomic assignments (**right**) of open reading frames (ORFs) encoding processes of: (**1**^**st**^**row**) dissimilatory sulfur metabolism; (**2**^**nd**^**row**) redox cofactors involved in the electron transfer chain; (**3**^**rd**^**row**) hydrogenases involved in the use of molecular hydrogen and electron-bifurcation complexes. The full list of enzymes and their gene abbreviations is available as supplement (Supplementary Table S1).

Sulfate taken up from the environment is activated in the cell by ATP and sulfate adenylyltransferase (*sat*) to form adenosine-5′-phosphosulfate (APS). APS is then reduced to sulfite by *apr* genes with electrons transferred from a membrane-bound quinone complex, and further reduced to H_2_S via *dsr* genes [43]. Genes encoding proteins for dissimilatory reduction of sulfate (*apr*), sulfite (*dsr*, *asr*), and thiosulfate/polysulfide (*phs*/*psr*) are few, or even negligible. They are mainly assigned to Chloroflexota, Desulfobacterota, Nitrospirota (including Thermodesulfovibrionia), and Firmicutes. In comparison, protein-encoding genes for respiratory reduction of elemental sulfur (S^0^) and polysulfide (*hyd*), and reversible sulfide dehydrogenase (*sud*), are abundant in metagenomes and almost exclusively assigned to Bathyarchaeia (Fig. 3). The output of FeGenie [37] indicates that, while metabolic potential for iron acquisition, storage and regulation is widespread in metagenomes, iron reduction is only found in Acidobacteriota and Nitrospirota (Supplementary Fig. S11). In the absence of clear indication for dissimilatory iron reduction [53], we searched for ORFs encoding redox enzymes (*trx*, *rbx*, *grx, fdx*) functioning as cofactors in the electron transfer chain and as potential ferric iron reductases. Although not ferric iron-specific, these cofactors that maintain reducing conditions in the cell can be coupled to membrane-bound, flavin-based assimilatory ferric iron reductases [54]. However, the abundance of these ORFs, which are common across Bathyarchaeia, Halobacterota, Chloroflexota, and Desulfobacterota, does not exhibit depth-dependent variations (Fig. 3). Further, ORFs encoding specific hydrogenases (*frh*, *mvh*, *hdr*) indicate that metabolic potential for energy-conserving use of molecular hydrogen [38] is present in several phyla (Thermoplasmatota, Euryarchaeota, Desulfobacterota, Firmicutes), but highest in Bathyarchaeia and Chloroflexota populations. Flavin-based electron bifurcation complexes were identified either solely in specific Bacteria (*Rnf*, i.e. sodium pump), or commonly across both Archaea (Bathyarchaeia, Halobacterota, Thermoplasmatota) and Bacteria (*Nuo*, i.e. proton pump). Detection of membrane-bound *Ech* hydrogenase was minor (Fig. 3).

**Figure 4:**
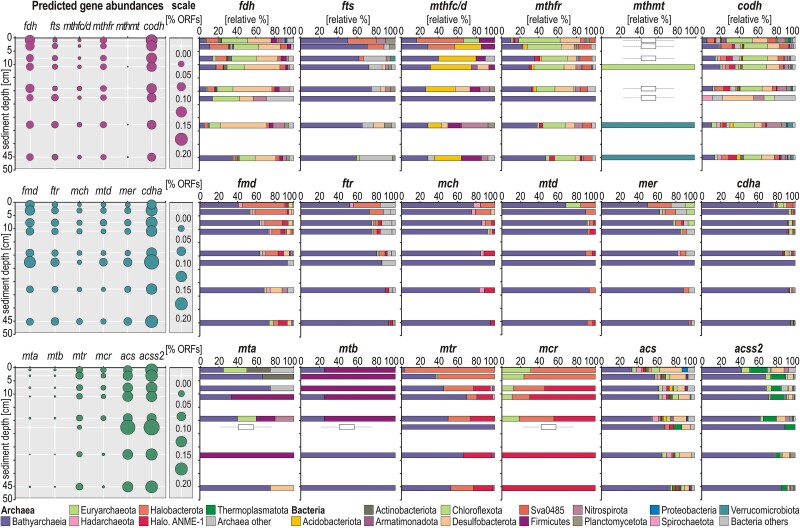
**Relative abundances of functional marker genes related to the Wood-Ljungdahl pathway and their corresponding taxonomic assignments.** Relative abundances (**left**) and taxonomic assignments (**right**) of open reading frames (ORFs) encoding metabolic steps involved in: (**1**^**st**^**row**) the carbonyl-branch of the Wood-Ljungdahl pathway; (**2**^**nd**^**row**) the methyl-branch of the Wood-Ljungdahl pathway; (**3**^**rd**^**row**) methytransferases and methane release from the cell, and acetate assimilation into biomass. The full list of enzymes and their gene abbreviations is available as supplement (Supplementary Table S1).

With regards to the W-L pathway [36, 55], ORFs encoding enzymes for the carbonyl- and methyl-branch are consistently assigned to Bacteria (Chloroflexota, Desulfobacterota, Nitrospirota, Planctomycetes) and Archaea (Bathyarchaeia, Nitrososphaeria, Euryarchaeota, Hadarchaeota, Halobacterota). Bathyarchaeia show high metabolic potential for both a partial carbonyl-branch and complete methyl-branch (Fig. 4). We identified methyltransferases related to different carbon sources, i.e. methanol (*mta*), methylamines (*mtb*) and CO_2_ (*mtr*), with a higher detection of *mtr*-related proteins whose taxonomic assignments correspond mainly to Bathyarchaeia. Metabolic potential to use methylthiols (*mts*) was not identified. ORFs encoding *mcr* proteins, whose subunit A catalyzes methane release from the cell or its anaerobic consumption, are assigned to Euryarchaeota, Halobacterota and ANME-1 (Fig. 4). Phylogenetic analysis of *mcr* amino acid sequences confirmed that none of the *mcrA*-encoding ORFs are assigned to Bathyarchaeia (Supplementary Fig. S12). *RuBisCO*, *fae* and *deoA* genes are assigned to Bathyarchaeia and Hadarchaeota. These enzymes enable anaerobic archaea to disproportionate formaldehyde (*fae*), sustain *RuBisCO* type III carboxylase activity via AMP phosphorylase (*deoA*), and divert riboses to central carbon metabolism at low energy levels [41, 42]. ORFs encoding proteins for the first step of the TCA and rTCA cycle (*cs*, *acly*) are minor (Supplementary Fig. S13).

### Adaptation to anoxic ferruginous sediments: genetic content of metagenome-assembled genomes

**Figure 5:**
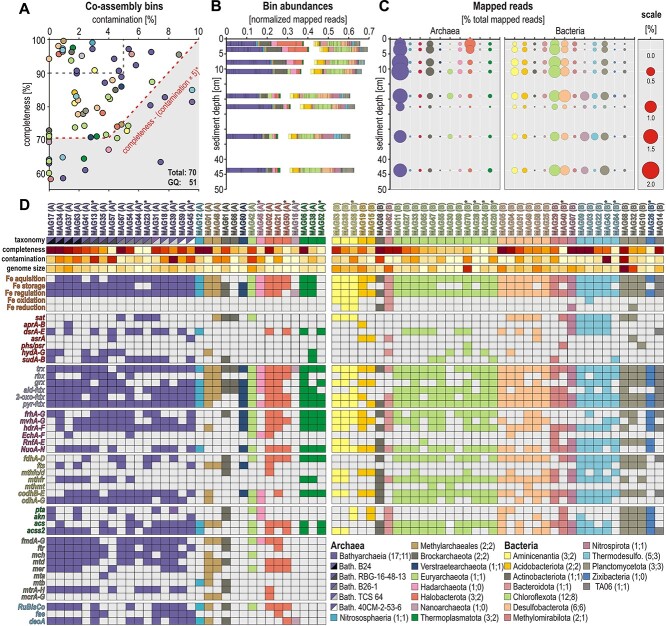
**Quality and abundances of metagenome-assembled genomes (MAGs) and specific metabolic features considered relevant to ferruginous conditions.** (**A**) MAGs assembled from 8 combined metagenomes plotted in terms of % contamination (x-axis) and completeness (y-axis). The 51 MAGs above the dotted line are considered of good to high quality. (**B**) Abundances of archaeal and bacterial bins in terms of normalized mapped reads against sediment depth. (**C**) Total mapped reads at the phylum/class level against sediment depth. (**D**) Presence/absence of functional marker genes in the 70 MAGs. MAGs marked with an asterisk (*) plot below the dotted line. The full list of enzymes and their gene abbreviations is available as supplement (Supplementary Table S1).

Genome bin abundances (normalized mapped reads) show that Bathyarchaeia, Chloroflexota and Desulfobacterota were the most abundant MAGs sequenced (Figs. 5B–5C). To validate the link between functions and taxonomy, we searched the same ORFs (Supplementary Table S1) in the genetic content of our 70 MAGs. According to FeGenie [37], metabolic potential to qualify as iron-reducing bacteria (FeRB) is only found in the MAGs of Aminicenentia (phylum Acidobacteriota) and Nitrospirota (Fig. 5D). Specific ORFs encoding proteins involved in dissimilatory reduction of sulfate to polysulfide (*sat*, *apr*, *dsr*, *asr*, *phs*/*psr*) are present in several MAGs of Chloroflexota, Desulfobacterota and Thermodesulfovibrionia (phylum Nitrospirota). Metabolic potential for dissimilatory sulfate and sulfite reduction (i.e. *aprAB, dsrAB*) was confirmed by phylogenetic analysis of *apr* and *dsr* amino acid sequences (Supplementary Fig. S14), indicating that only specific MAGs of Desulfobacterota, Nitrospirota (class Thermodesulfovibrionia), and Acidobacteriota qualify as potential SRB. This analysis also shows that several MAGs of Chloroflexota include the *dsrC* and *dsrE* subunits, which are relevant to cellular reduction and assimilation of sulfur [56], whereas 6 MAGs of Bathyarchaeia include the *dsrE* subunit, only. In addition, predicted ORFs related to respiratory reduction of polysulfide with reversible sulfide dehydrogenase (*hyd, sud*) are mainly present in MAGs of Bathyarchaeia (Fig. 5D). ORFs encoding redox cofactors involved in the electron transport chain (*trx*, *rbx*, *grx*, *ald- fdx*, *2-oxo-fdx*, *pyr-fdx*) were detected in almost all the MAGs of Bathyarchaeia, Chloroflexota, and Desulfobacterota (Fig. 5D). Potential use of molecular hydrogen via hydrogenases (*frh*, *mvh*, *hdr*) is highest in Bathyarchaeia, Chloroflexota, Desulfobacterota and Aminicenantia (phylum Acidobacteriota), which could be combined with electron bifurcation for energy conservation via the *Rnf* complex (i.e. sodium pump) in Aminicenentia, Thermodesulfovibrionia and Planctomycetota (Fig. 5D), and the *Ech* and mainly *Nuo* complexes (i.e. proton pumps) in Bathyarchaeia, Halobacterota, Thermoplasmatota, and diverse Bacteria (Fig. 5D).

Altogether, the genetic content of MAGs shows that several bacterial phyla (i.e. Desulfobacterota, Nitrospirota, Acidobacteriota) could combine a certain degree of dissimilatory iron and sulfate reduction with the carbonyl-branch of the W-L pathway and electron bifurcation. Metabolic potential to convert acetate into biomass (*acs*, *acss2*) is widely present, whereas acetate conversion to acetyl-CoA (*pta*, *akn*) via substrate-level phosphorylation (SLP) is a less common feature (Fig. 5D; Supplementary Fig. S13). In contrast, Chloroflexota exhibit metabolic capability to acquire iron and sulfur in a fermentative acetogenic lifestyle (Fig. 5D). ORFs identified in the MAGs of Bathyarchaeia, Methylarchaeales, and Halobacterota indicate metabolic potential for CO_2_ fixation via the complete methyl-branch W-L pathway. ORFs encoding *codh* and *cdhA* in Bathyarchaeia, Chloroflexota, Desulfobacterota and Nitrospirota (Fig. 5D) reveal metabolic potential for CO_2_ fixation and energy production in (homo)acetogenic W-L pathway. Although we identified 16S rRNA genes assigned to several putative methanogens (Supplementary Fig. S1), the presence of *mcrA* genes in our MAGs is restricted to the order Methylarchaeales [50], Methanomicrobiales and ANME-1 (Supplementary Fig. S12). Despite the group BA1 and BA2 being known methanogens among Bathyarchaeia [50, 57], we did not identify *mcr*-related ORFs in Bathyarchaeia, neither were they assigned to the BA2 or BA2 group (Supplementary Figs. S9 and S12). In contrast, we identified metabolic potential to produce and assimilate acetate (*acs*, *acss2*) via the methyl-branch of the W-L pathway (Fig. 5D), the bathyarchaeal groups B24 and B26-1 being able to convert acetyl-CoA into acetate (*pta*, *akn*), or its reverse acetoclastic function, for additional energy production. Pangenome analysis carried out on the 17 MAGs of Bathyarchaeia with 22 GTDB representative MAGs argues for a genetic content that indicates they are (homo)acetogens (Supplementary Fig. S10), not methanogens [58–60]. The carbohydrate-active enzyme analysis (CAZymes) integrated in anvi'o confirms that the diversity of CAZymes available to Bathyarchaeia for degrading complex organic polymers is high, in particular in group B24 (Supplementary Fig. S15).

Further, the presence/absence of genes encoding methytransferases (*mta*, *mtb*, *mtr*) in archaeal MAGs indicate that Bathyarchaeia can use CO_2_ and methylated compounds as carbon sources [61]. Thus, these 17 MAGs of Bathyarchaeia encode a partial carbonyl-branch and complete methyl-branch of a methylotrophic acetogenic W-L pathway [36, 62, 63], with metabolic capability to produce, assimilate or convert acetate and formate from CO_2_. Finally, although we identified ORFs encoding *fae* proteins which enable condensation and dissimilation of formaldehyde with tetrahydromethanopterin [42], those encoding *RuBisCO* in these MAGs correspond to the archaeal type III, which does not participate in autotrophic CO_2_ fixation but in adenosine 5′-monophosphate (AMP) metabolism with *deoA* genes that divert riboses to support glycolysis at low energy levels [41].

## Discussion

In Lake Towuti’s anoxic ferruginous sediments, FeRB and SRB exhibited a certain degree of metabolic versatility, allowing them to switch from respiration of reactive ferric minerals and SO_4_^2−^ to fermentation (Figs. 3-5). After pore water depletion in electron acceptors, we expected that FeRB and SRB would be gradually replaced by primary (i.e. acidogens) and secondary (i.e. acetogens) fermenters [64]. Still, the Acidobacteriota (class Aminicenantia), which are predicted to act as primary degraders of buried OM during acidogenesis, fermenting proteinaceous substrates and different sugars producing hydrogen and acetate as by-products [65], were identified as potential FeRB (Fig. 5D). Redox cofactors (*trx*, *rbx*, *grx*, *fdx*) are not ferric iron-specific enzymes, but also allow for the reduction of other organic or inorganic compounds [54], which implies a certain degree of metabolic versatility among FeRB and SRB. Thus, we suggest that microbial reduction of mineral ferric iron could proceed at slow rates [17] via redox cofactors, enhancing hydrogenase (*frh*, *mvh*, *hdr*) activity [66] alongside electron bifurcation (*Rnf*, *Nuo*) and fermentation (Fig. 5). Similarly, the Nitrospirota (class Thermodesulfovibriona) and the Desulfobacterota, while being SRB (*dsrAB*, *aprAB*), were metabolically capable of energy conservation via a partial hydrogenotrophic and acetoclastic (*pta*, *akn*) oxidative W-L pathway. Several redox cofactors (*trx*, *rbx*, *grx*), which are hypothesized to mitigate oxidative stress during sulfate reduction and, by extension, under sulfate-poor ferruginous conditions [67–69], could also function with hydrogenases (*mvh*, *hdr*) and electron bifurcation complexes (*Ech*, *Rnf*, *Nuo*) to provide redox-conserving energy. As SRB are known to be fast degraders of labile OM [43, 70], the aforementioned metabolic traits suggest that alternative electron donors may be used below the depth of sulfate depletion, allowing them to persist as fermenters outside their geochemical niche (Fig. 5D).

During fermentation stages, C1 and C2 compounds, like formate, methanol, CO^−^, and acetate, are remineralized into methanogenic substrates or taken up by non-syntrophic anaerobes [64, 71]. Among those, the Hadarchaeota became increasingly abundant below 20 cmblf (Fig. 2B). This phylum exhibits metabolic traits centered on H_2_ and CO^−^ oxidation combined with a partial W-L pathway [72]. Similarly, the Chloroflexota (class Dehaloccoidia) have a fermentative lifestyle with a reductive W-L pathway to facilitate cell carbon synthesis from acetate (*acs*, *acss2*). The fact that these clades remained relatively abundant down to 50 cmblf suggested that (homo)acetogens could outperform methanogens in the uptake of C1 and C2 metabolites [73] in either an oxidative or reductive W-L pathway [61, 62]. The methanogens identified (Supplementary Fig. S1) appeared to be mostly methylotrophic (Methanomassiliicoccales, Methanofastidiosales, Methanomethyliales), with fewer taxa related to hydrogenotrophic methanogenesis (e.g. Methanomicrobiales) and methanotrophs (ANME-1). We detected *mcrA* genes in three specific MAGs (Fig. 5D; Supplementary Fig. S12) that were concomitant either with *mta* and *mtb* genes (i.e. methylotrophic) in *Candidatus* Methylarchaeales [50], or with *mtr* genes (i.e. hydrogenotrophic) in Methanomicrobiales [63, 74]. Despite the relatively low abundance of methanogens, methane accumulated to 550 μM at 50 cmblf, with upward diffusion apparently feeding ANME-1 populations [10, 11] in the upper 20 cmblf (Figs. 2A-2B). We further suspected that C1 compounds and molecular hydrogen sustaining methylotrophic/hydrogenotrophic methanogenesis might be produced during OM fermentation by Bathyarchaeia [57, 75], which constitute the majority of the microbial assemblage below 20 cmblf (Fig. 2B).

Bathyarchaeia can utilize a wide range of labile and refractory organic compounds, e.g. proteins, carbohydrates, volatile fatty acids, short-chain alkanes, methylated compounds, cellulose, lignin [57, 76]. At the same time, they also use multiple C1 compounds [49] and drive CO_2_ fixation with molecular hydrogen to convert it to acetyl-CoA and acetate via the W-L pathway. The question arose as to whether Bathyarchaeia play an active role in syntrophic interactions in lake sediments, as already proposed for paddy fields [77, 78], peatlands [58, 75], and estuaries [79, 80], or whether they are linked to the plethora of reactive iron minerals (>10 weight %) and scarcity of sulfate in Lake Towuti, or simply to OM types derived from tropical vegetation in the catchment [3, 11]. In the maar Potrok Aike [47], Bathyarchaeia were mainly found in deep layers composed of organic-poor detrital mafic material while Atribacteriota, Chloroflexota, Aminicenentia formed the predominant subsurface biosphere in methane-rich sediments. In sulfate-rich (~2 mM) Lake Cadagno [81], Bathyarchaeia were abundant at shallow depths around the sulfate–methane transition and prevailed below the depth of sulfate depletion. Overall, the geographical distribution of Bathyarchaeia has been proposed to result from salinity gradients and OM types, enabling to trace their orders in terms of sediment provenance [49, 57, 75, 82], i.e. soils (40CM-2-53-6), groundwater (RBG-16-48-13), hot springs (B24), estuaries (TCS64), cold marine sediments (B26-1), and hydrothermal vents (EX4484–135, B25). Apart from hydrothermal vents, Lake Towuti’s ferruginous sediments harbored groups of all origins (Supplementary Figs. S9 and S10), suggesting that ferruginous geochemical conditions constitute a preferential niche for Bathyarchaeia.

### Bathyarchaeia thrive below the sulfate reduction zone

Low but sustained SRR values (≤ 2 nmol × cm^−3^ × day^−1^) coincided with the minor detection of protein-encoding genes (*asr, phs/psr*) involved in the reduction of sulfur intermediates (SO_3_^2−^, S_2_O_3_^2−^, S_N_^2−^) in the presence of few ANMEs and methanogens (Figs. 2-3), suggesting that cryptic sulfur cycling and AOM processes occurred across the sulfate–methane transition [43, 83]. Below the active sulfate reduction zone, Bathyarchaeia prevailed in the fermentative zone (Fig. 2B). Several of their MAGs revealed metabolic capabilities either in sulfide dehydrogenase (*sud*) activity [84] with polysulfide (*hyd*) as electron acceptor [85, 86], or reverse in fermentation coupling heterodisulfide reductase (*hdr*) with electron bifurcation (*Ech*, *Nuo*), or both [70]. By metabolizing sulfide into sulfur, and sulfur into polysulfide via rubredoxin [84, 85], and assimilating persulfide with the *dsrE* protein as acceptor [56, 87], Bathyarchaeia feature a redox complex offering an alternative metabolic pathway that makes elemental sulfur disproportionation energetically more favorable. Soluble polysulfides can effectively scavenge hydrogen sulfide (H_2_S) to form elemental sulfur (S^0^) chains of up to eight atoms [86], which allows continuous sulfide removal without the need for sulfur disproportionation to sulfate. Thus, microbial cycling of sulfur intermediates via polysulfides could account for residual SRR measured below 5 cmblf. Although polysulfide is more soluble than sulfide in the pore water [86], it reacts abiotically with OM to produce poorly reactive sulfurized compounds [43, 88]. In the absence of pore water polysulfide, we hypothesize that this function could be reversed towards fermentation.

In the fermentative zone, Bathyarchaeia could rely on their heterotrophic capabilities to perform fermentation of polysaccharides and proteins [57, 89], while able to couple the use of molecular hydrogen (*frh*, *mvh*, *hdr*) with electron bifurcation (*Ech*, *Nuo*) in the complete methyl-branch of the W-L pathway [90, 91] to convert CO_2_ into acetyl-CoA under low-energy conditions [92]. In the absence of *mcrA* genes (Supplementary Fig. S12), none of the bathyarchaeal groups identified (B24, B26-1, 40CM-2-53-6, TCS64, RBG-16-48-13) corresponded to those performing methanogenesis (BA1, BA2). Instead, their methanopterin-based W-L pathway appeared to be partially methylotrophic [49, 93], using methanol (*fae*, *mta*), methylamines (*mtb*), formate (*fdh*) and CO_2_ (*codh*, *mtr*) as diverse C1 compounds, to produce energy and assimilate acetate into biomass (*acs*, *acss2*). Yet, the groups B24 and B26-1 [90] qualified as full homoacetogens as they exhibited metabolic capability to catalyze the conversion of acetyl-CoA into acetate (*pta*, *akn*). While acting as an electron sink in heterotrophic acetogenesis (Fig. 5D), their partial carbonyl-branch (i.e. bacterial type) combined with SLP could participate in tetrahydrofolate interconversions of C1 and C2 compounds, mostly from CO_2_ to formate and acetate [49, 93–95]. Similar to termite guts [96], such W-L pathway combined with complex carbohydrate degradation (Supplementary Fig. S15) could support syntrophic associations with methylotrophic methanogens.

### Potential metabolic features inherited from ancient ferruginous ecosystems

Bathyarchaeia are postulated to have arisen at hydrothermal vents [93, 94] and diversified in ferruginous and euxinic environments during the Proterozoic [49, 57], perpetuating several primitive sulfur-based and energy-conserving metabolic features of methylotrophic archaea [59]. Among these, cryptic sulfur cycling coupled with CO_2_ fixation via the W-L pathway [97] are presumedly prominent features of primeval microbial life in ancient ferruginous systems [93]. Similarly, dissimilatory iron reduction is thought to be one of the oldest energy-generating processes [54] that may have evolved on Earth from catalytic minerals into electron-transporting metalloenzymes [66]. In Earth’s early oceans, the metabolic capability to access limited resources in bioavailable sulfur, metals and OM appear to have played a key role in driving enzymatic reactions relevant to the W-L pathway at the origin of life [36, 66].

By analogy, in modern ferruginous sediments, Bathyarchaeia prevailed below the sulfate reduction zone upon depletion of reactive iron minerals and labile organic substrates. Their MAGs included metabolic machinery for respiration of soluble polysulfides [87] and assimilation via the *hdr*-complex, *dsrE* and rubredoxin, inferring an ability to harness reactive sulfur species [98] via polysulfide cycling. The presence of an archaeal type *RuBisCO* III pathway with genetic potential to recycle AMP, methanol and formaldehyde (*deoA*, *fae*) further suggested that phosphoriboses (i.e. necromass) could originally be fermented under organic-lean, high CO_2_ conditions [41, 99]. By taking advantage of both a partial catabolic (carbonyl-branch) and complete anabolic (methyl-branch) W-L pathway, Bathyarchaeia developed a mixotrophic lifestyle as methylotrophic/acetogenic fermenters capable of fixing CO_2_ [61, 95]. Their metabolic potential to degrade complex carbohydrates and proteins [82] grafted onto such redox-conserving homoacetogenic W-L pathway may have led to excess production of C1-C2 compounds and molecular hydrogen during OM fermentation, thereby developing close (syntrophic) interactions with methylotrophic methanogens [50, 52] and non-methanogens [51] in modern ecosystems.

## Supplementary Material

Supplementary_Material_final_ycae112

## Data Availability

All raw sequencing data are publicly available on the European Nucleotide Archive (ENA) under project accession number PRJEB66721 (https://www.ebi.ac.uk/). The geochemical data is publicly available as PANGAEA dataset #861437 (https://doi.pangaea.de/). All scripts and instructions to conduct taxonomic and functional annotations are posted on GitHub (github.com/williamorsi/MetaProt-database).
