# Supplementary material for: Metabolic features that select for Bathyarchaeia in modern ferruginous lacustrine subsurface sediments
Source: ISME Commun. 2024 Sep 14;4(1):ycae112. doi: 10.1093/ismeco/ycae112 (PMC11631310; doi:10.1093/ismeco/ycae112)
Supplement: Supplementary_Material_final_ycae112 [file supplementary_material_final_ycae112.pdf]

Supplementary Material

Metabolic features that select for Bathyarchaeia in modern ferruginous lacustrine subsurface sediments

Fatima Ruiz-Blas, Alexander Bartholomaüs, Sizhong Yang, Dirk Wagner, Cynthia Henny, James M. Russell, Jens Kallmeyer and Aurèle Vuillemin

Content: Supplementary Figures (15); Supplementary Tables (4); Supplementary Methods, Supplementary References (35).

Supplementary Figures

- **Supplementary Figure S1.** Phylogenetic tree of 16S rRNA genes assigned to putative methanogens.
- **Supplementary Figure S2.** Phylogenetic tree of 16S rRNA genes assigned to putative sulfate-reducing bacteria among Firmicutes and Nitrospirota.
- **Supplementary Figure S3.** Phylogenetic tree of 16S rRNA genes assigned to putative sulfate-reducing bacteria among Desulfobacterota.
- **Supplementary Figure S4**. Phylogenetic tree of 16S rRNA genes assigned to Chloroflexota.
- **Supplementary Figure S5**. Phylogenetic tree of 16S rRNA genes assigned to Bathyarchaeia.
- **Supplementary Figure S6.** Bar charts of 16S rRNA genes assigned to putative methanogens and sulfate-reducting bacteria.
- **Supplementary Figure S7.** Non-metric multidimensional scaling (NMDS) analyses based on a total of 4 559 ASVs and 138 243 ORFs.
- **Supplementary Figure S8.** Phylogenetic tree of assembled 16S rRNA genes retrieved from the MAGs.
- **Supplementary Figure S9.** Phylogenetic tree based on 16 concatenated ribosomal protein sequences for the 17 metagenome-assembled genomes (MAGs) assigned to Bathyarchaeia in this study with 286 representative MAGs from the GTDB databes as reference.
- **Supplementary Figure S10.** Pangenomic analysis of the 17 metagenome-assembled genomes (MAGs) assigned to Bathyarchaeia with 22 GTDB representative MAGs as reference.
- **Supplementary Figure S11.** Heatmaps of metabolic potential associated with iron processes based on the FeGenie pipeline.
- **Supplementary Figure S12.** Relative abundances of additional functional marker genes and their corresponding taxonomic assignments.
- **Supplementary Figure S13.** Phylogenetic tree of methyl-coenzyme M reductase proteins.
- **Supplementary Figure S14.** Phylogenetic tree of dissimilatory sulfite reductase and adenylylsulfate reductase proteins.
- **Supplementary Figure S15.** Heatmap of the CAZyme analysis of the 17 metagenome-assembled genomes (MAGs) assigned to Bathyarchaeia.

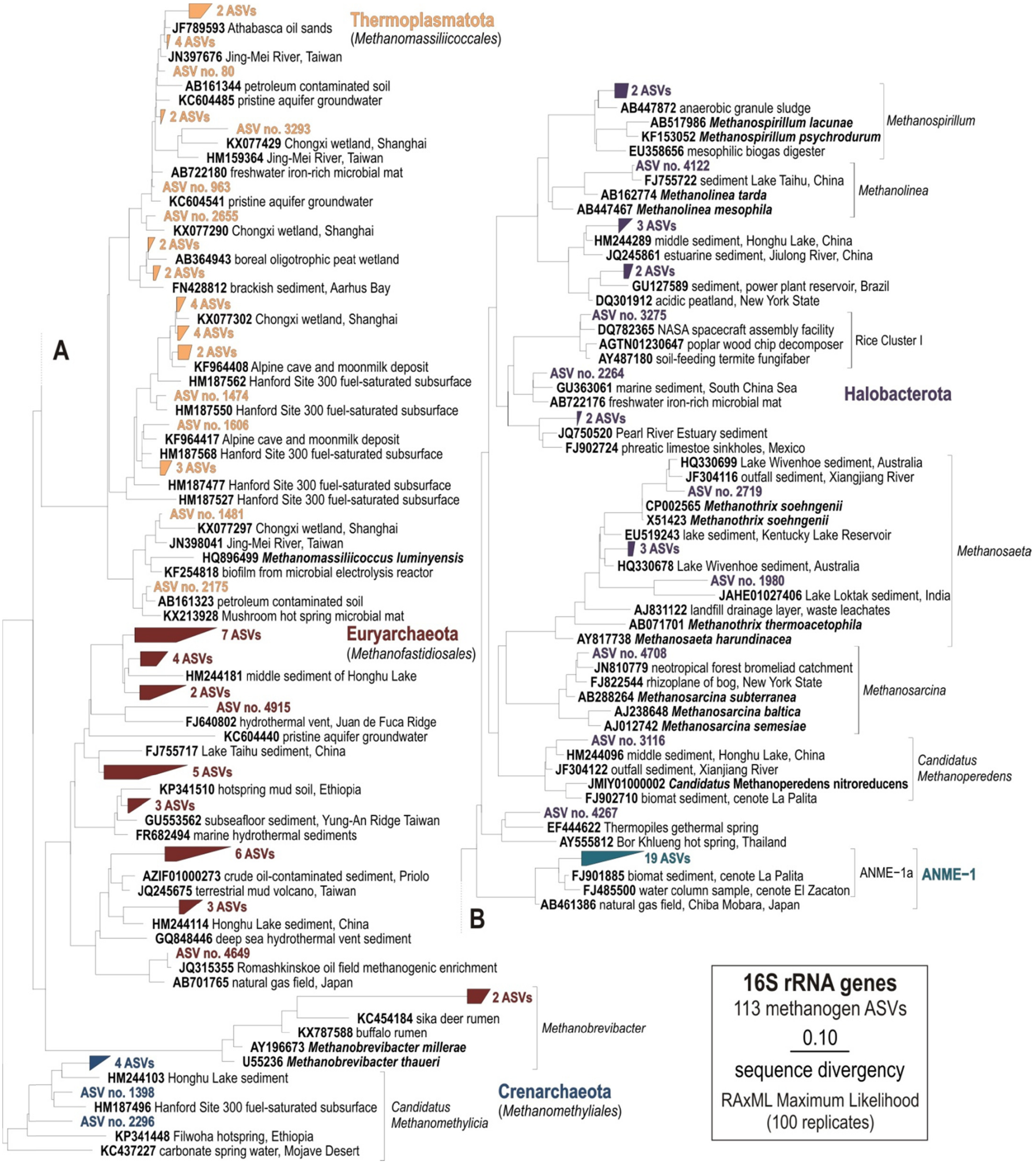

Supplementary Figure S1. Phylogenetic tree of 16S rRNA genes (V4 hypervariable region) for amplicons sequenced in this study taxonomically assigned to putative methanogens and closely related taxa (113 ASVs). Boldface types signify cultivated species and sequence accession numbers to the SILVA database [1, 2]. The RAxML maximum Likelihood phylogenetic tree was calculated selecting the best tree among 100 replicates, using rapid bootstrap analysis and inserting partial 16S rRNA gene amplicons (500 bps) applying the ARB Parsimony algorithm with the bacterial and archaeal filters on ARB [3].

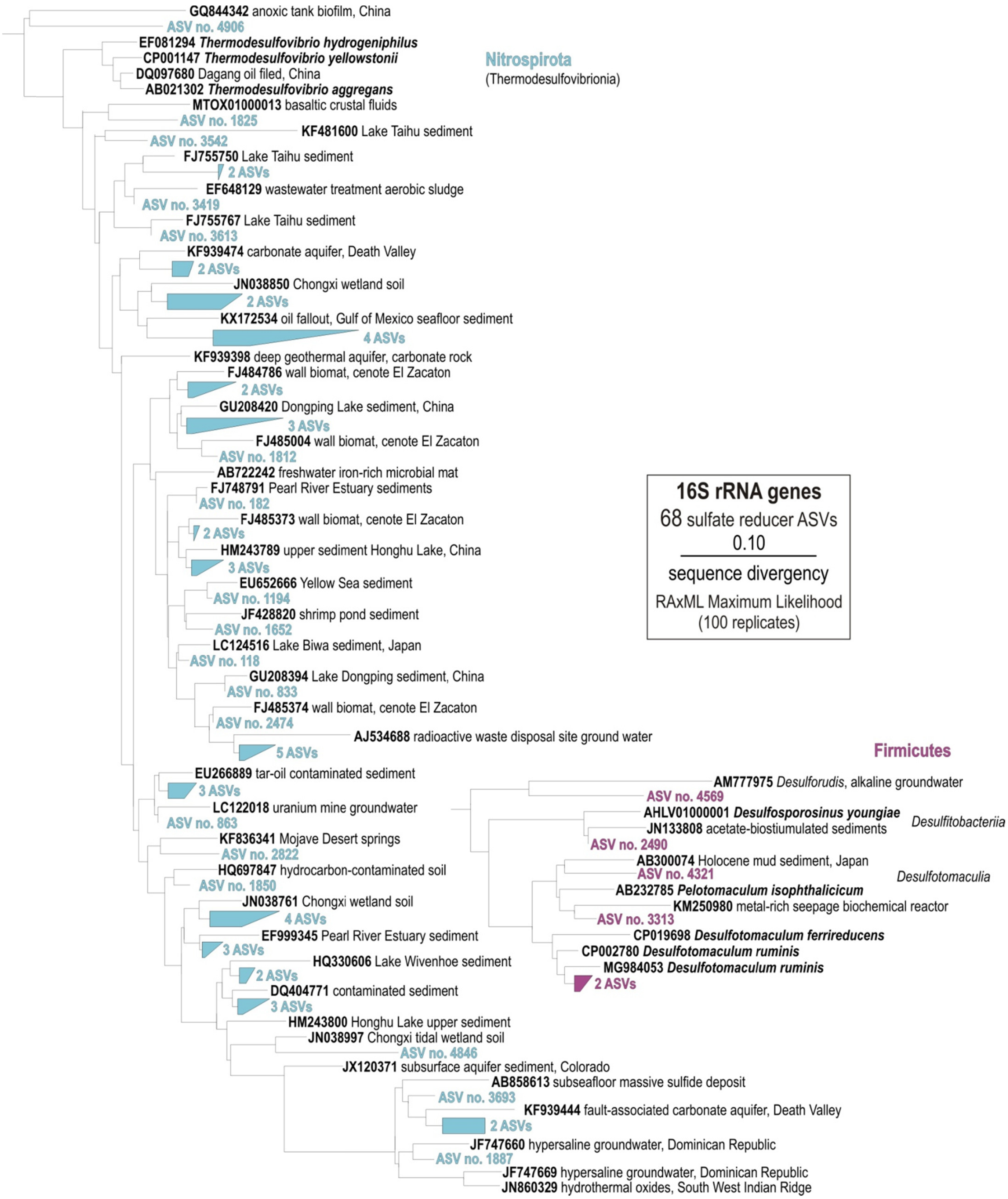

Supplementary Figure S2. Phylogenetic tree of 16S rRNA genes (V4 hypervariable region) for amplicons sequenced in this study taxonomically assigned to putative sulfate-reducing bacteria, and closely related taxa, among Firmicutes (6 ASVs) and Nitrospirota (62 ASVs). Boldface types signify cultivated species and sequence accession numbers to the SILVA database [1, 2]. The RAxML maximum Likelihood phylogenetic tree was calculated selecting the best tree among 100 replicates, using rapid bootstrap analysis and inserting partial 16S rRNA gene amplicons (500 bps) applying the ARB Parsimony algorithm with the bacterial and archaeal filters on ARB [3].

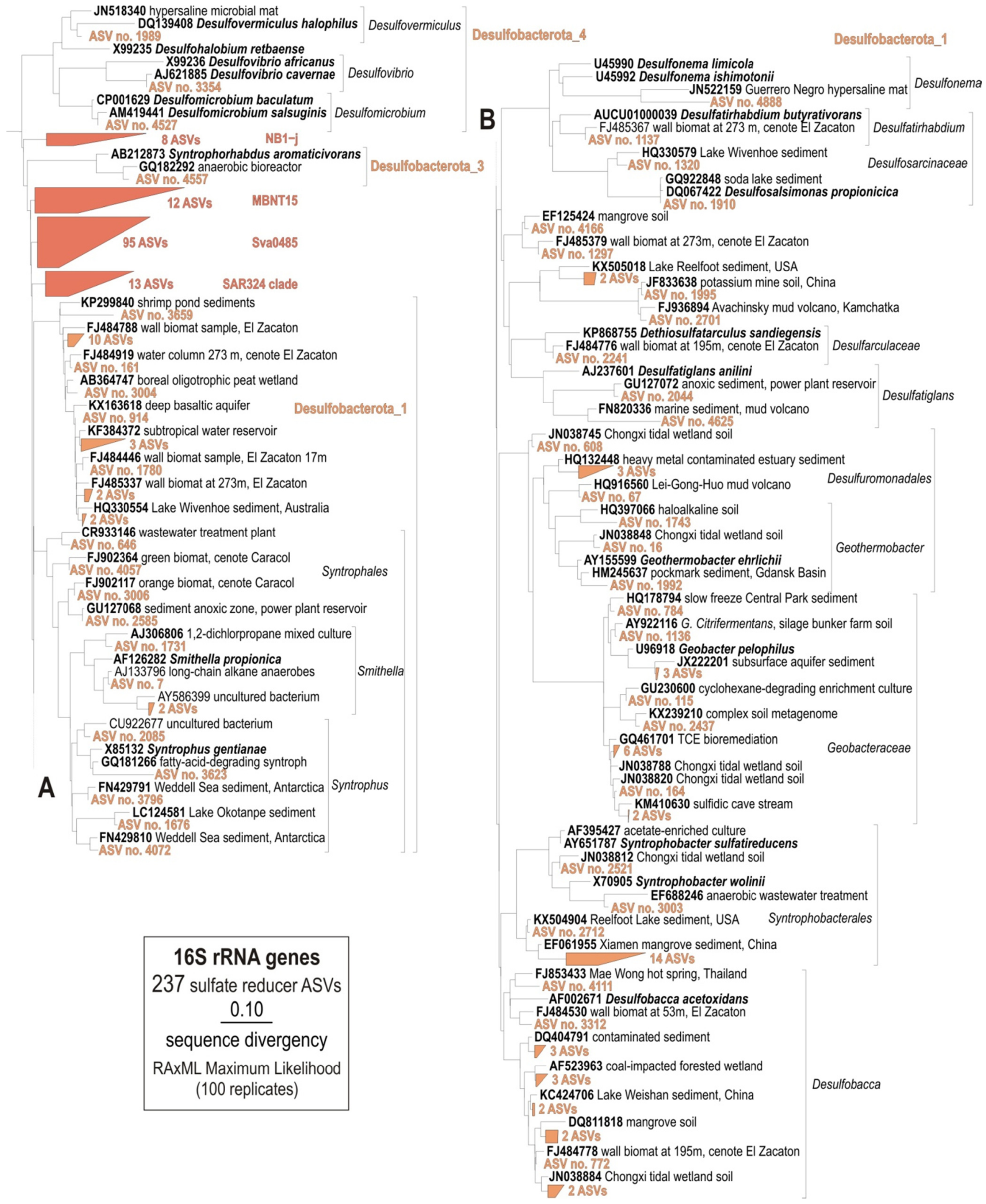

Supplementary Figure S3. Phylogenetic tree of 16S rRNA genes (V4 hypervariable region) for amplicons sequenced in this study taxonomically assigned to putative sulfate-reducing bacteria among Desulfobacterota (6 ASVs) and closely related clades (62 ASVs). Boldface types signify cultivated species and sequence accession numbers to the SILVA database [1, 2]. The RAxML maximum Likelihood phylogenetic tree was calculated selecting the best tree among 100 replicates, using rapid bootstrap analysis and inserting partial 16S rRNA gene amplicons (500 bps) applying the ARB Parsimony algorithm with the bacterial and archaeal filters on ARB [3].

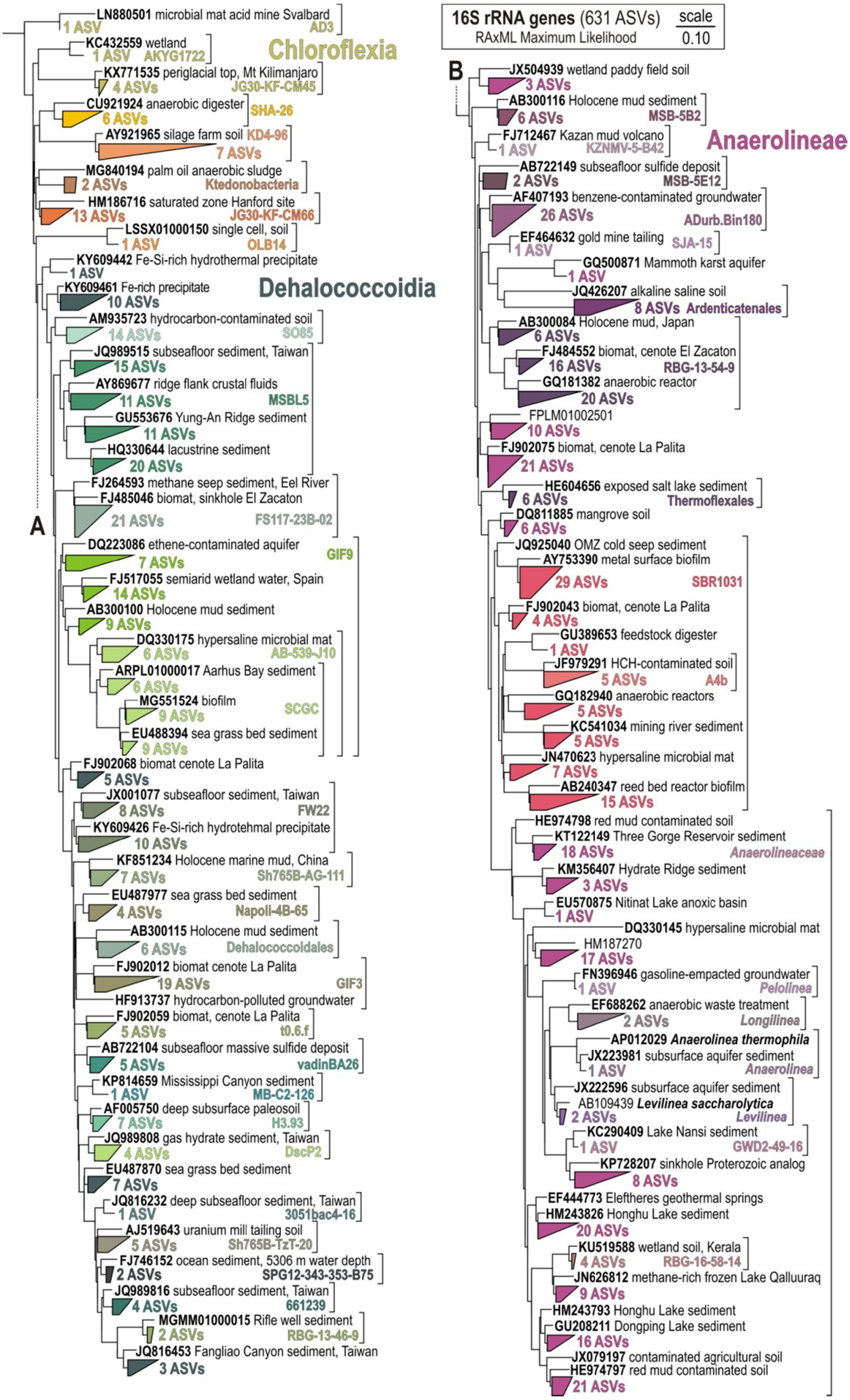

Supplementary Figure S4. Phylogenetic tree of 16S rRNA genes (V4 hypervariable region) for amplicons sequenced in this study taxonomically assigned to Chloroflexota (631 ASVs). Boldface types signify cultivated species and sequence accession numbers to the SILVA database [1, 2]. The RAxML maximum Likelihood phylogenetic tree was calculated selecting the best tree among 100 replicates, using rapid bootstrap analysis and inserting partial 16S rRNA gene amplicons (500 bps) applying the ARB Parsimony algorithm with the bacterial and archaeal filters on ARB [3].

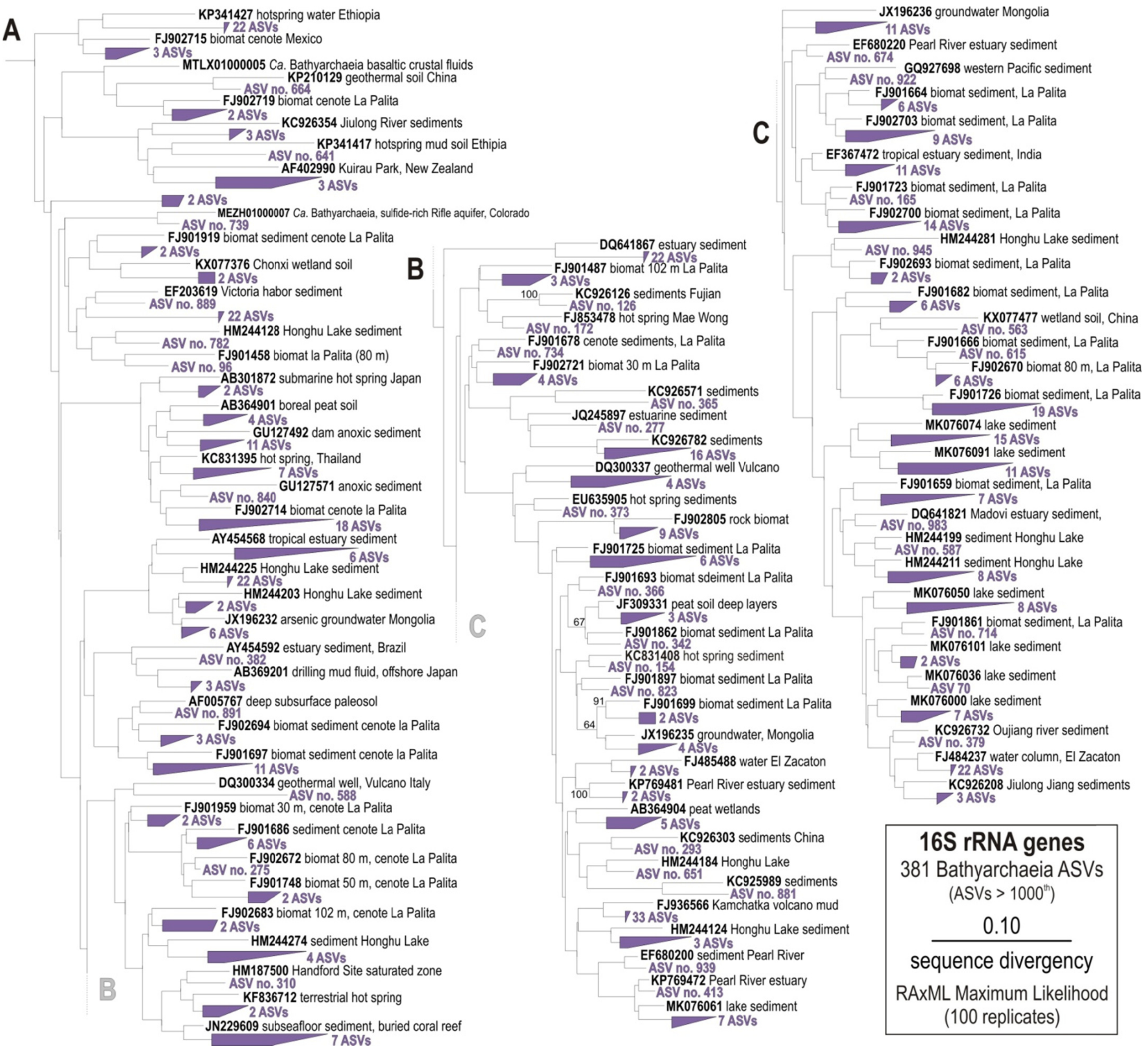

Supplementary Figure S5. Phylogenetic tree of 16S rRNA genes (V4 hypervariable region) for amplicons sequenced in this study taxonomically assigned to Bathyarchaeia (including 381 ASVs above the 1000th ASV; 965 Bathyarchaeia ASVs in total). Boldface types signify cultivated species and sequence accession numbers to the SILVA database [1, 2]. The RAxML maximum Likelihood phylogenetic tree was calculated selecting the best tree among 100 replicates, using rapid bootstrap analysis and inserting partial 16S rRNA gene amplicons (500 bps) applying the ARB Parsimony algorithm with the bacterial and archaeal filters on ARB [3].

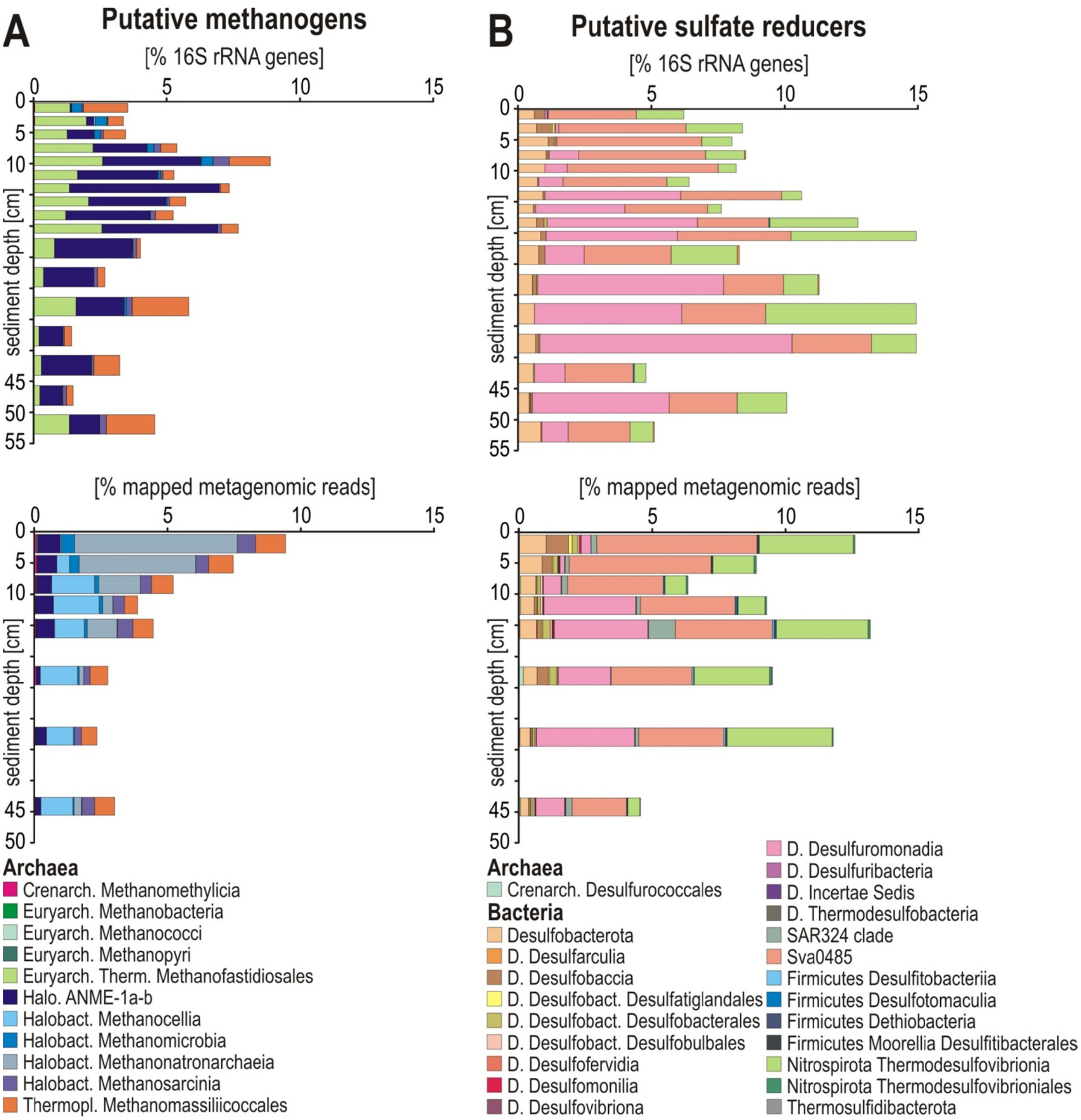

Supplementary Figure S6. Relative abundances of putative methanogens (**A**) and sulfate-reducing bacteria (**B**) based on taxonomic assignments of 16S rRNA genes (1st row) and metagenomic reads (2nd row) mapped to the SILVA database [1, 2].

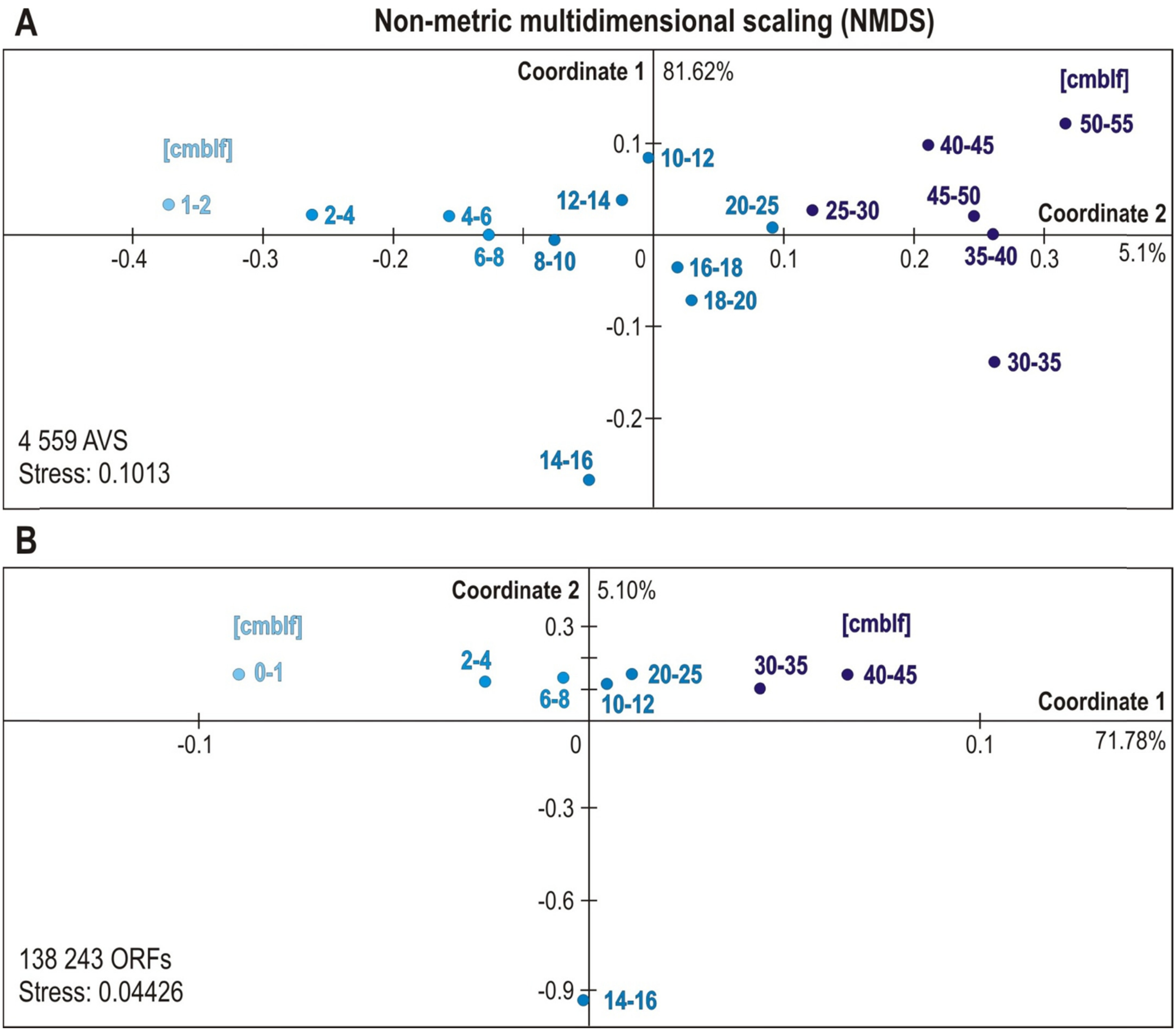

Supplementary Figure S7. (**A**) Non-metric multidimensional scaling (NMDS) calculated from 4,559 ASVs obtained across 18 samples. The coordinates 1 and 2 account for about 86 % of the variation explained (stress = 0.1013) and consistently distribute samples according to sediment depth (coordinate 1 = 81.62 %) and geochemistry (coordinate 2 = 5.13 %). (**B**) NMDS calculated from 138 243 Open Reading Frames (ORFs) obtained across 8 metagenomic libraries. The coordinates 1 and 2 account for about 77 % of the variation explained (stress = 0.04426) and distributes samples consistently according to taxonomic (A) and functional (B) diversity. The NMDS were calculated using Past v. 4.03 [4], applying the Bray-Curtis similarity index.

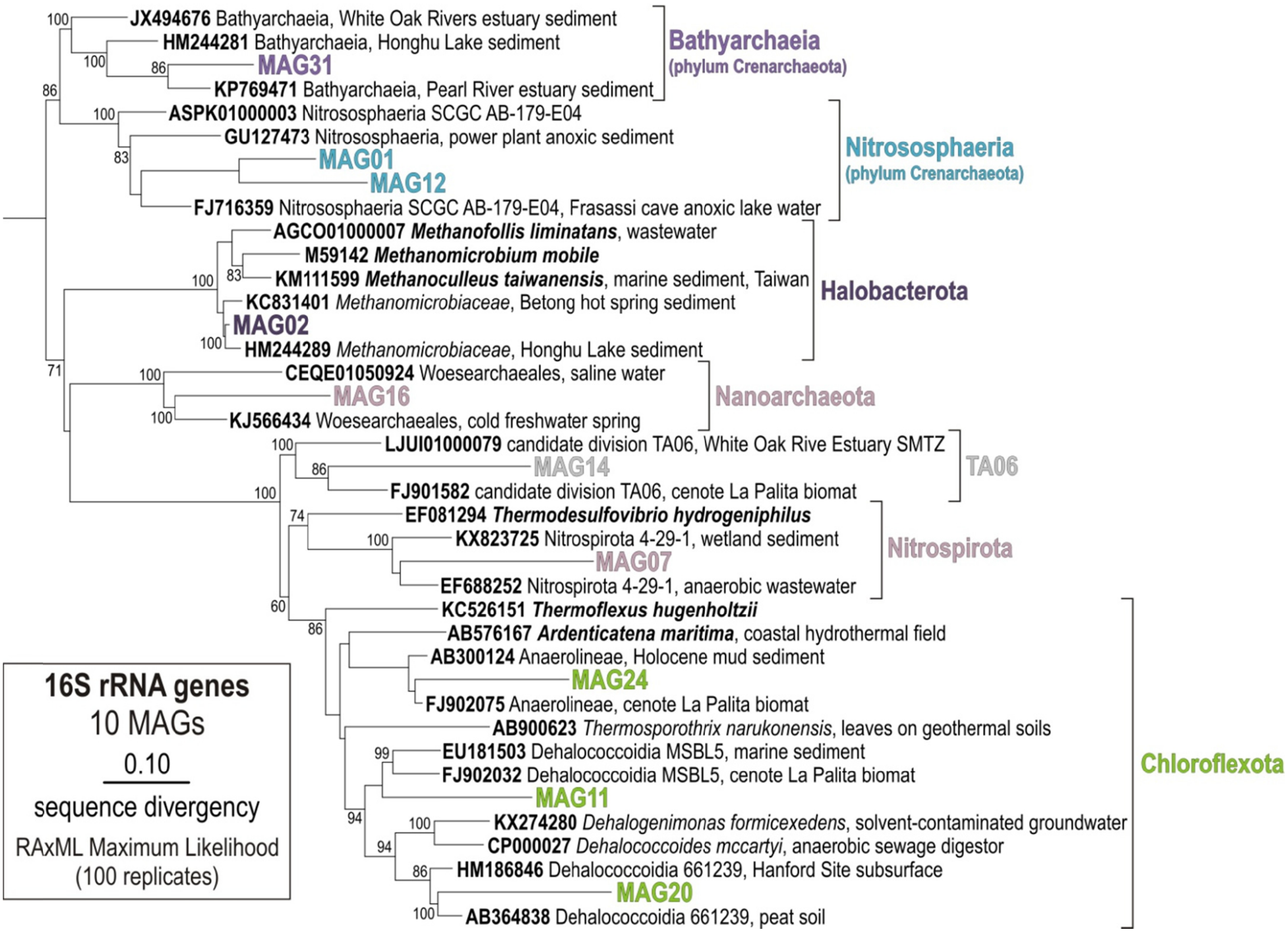

Supplementary Figure S8. Phylogenetic tree of assembled 16S rRNA genes extracted from the metagenome-assembled genomes (MAGs), providing confirmation of the GTDB taxonomy [5] assigned by the ATLAS pipeline [6]. Boldface types signify cultivated species and sequence accession numbers to the SILVA database [1, 2]. The RAxML maximum Likelihood phylogenetic tree was calculated selecting the best tree among 100 replicates, using rapid bootstrap analysis and inserting partial 16S rRNA gene amplicons (500 bps) applying the ARB Parsimony algorithm with the bacterial and archaeal filters on ARB [3].

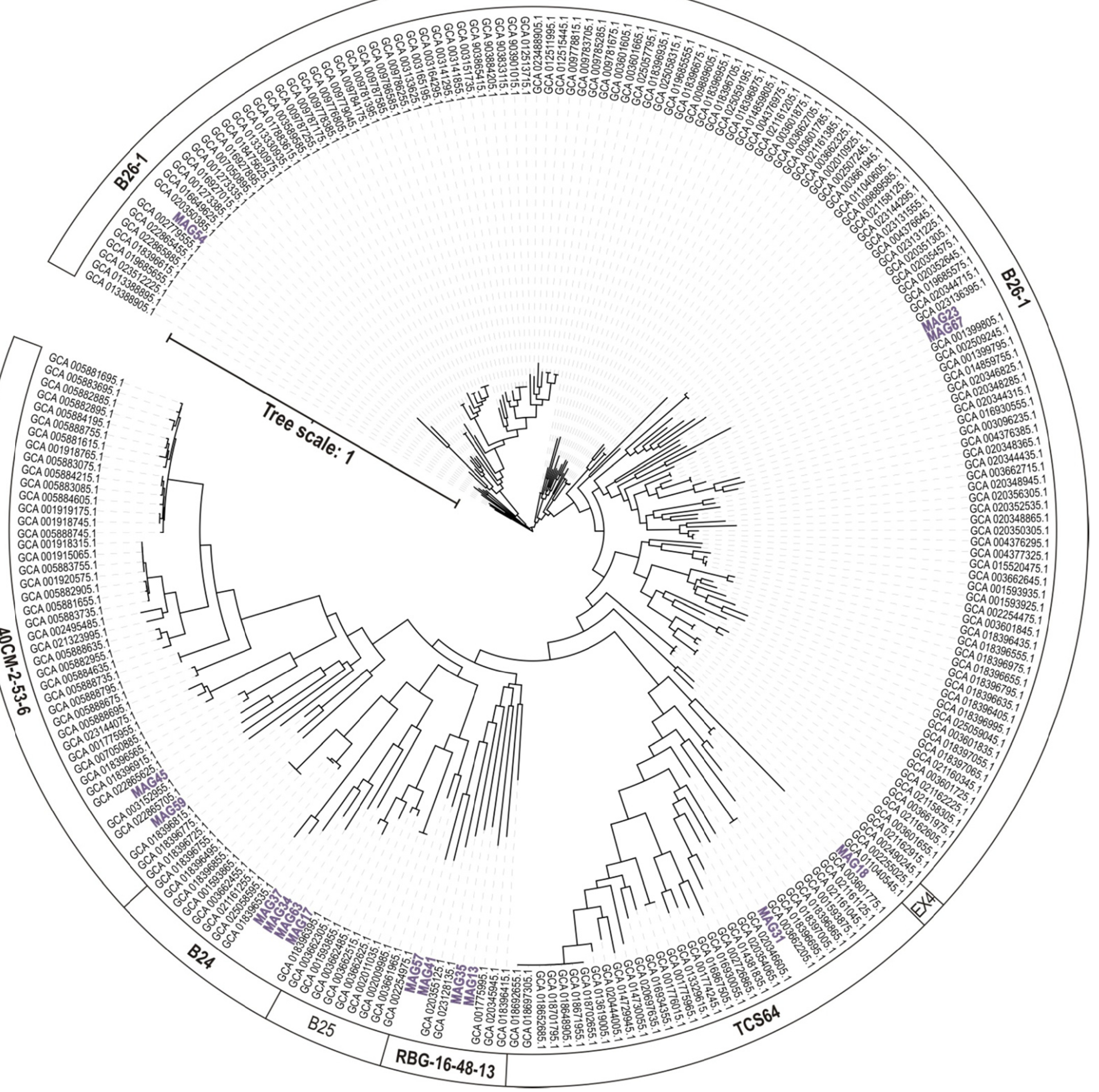

Supplementary Figure S9. Phylogenetic tree based on 16 concatenated ribosomal protein sequences extracted from all metagenome-assembled genomes (MAGs) assigned to Bathyarchaeia and representative MAGs (286) from the GTDB database [5] as references, using open scripts and published protocol [7, 8]. Tree visualization was achieved using iTOL v. 6 [9]. Sustainable cultivation is only available for Bathyarcheum tardum [10] while *Candidatus* Hecatella orcuttiae represents one whole representative metagenome-assembled genome [11].

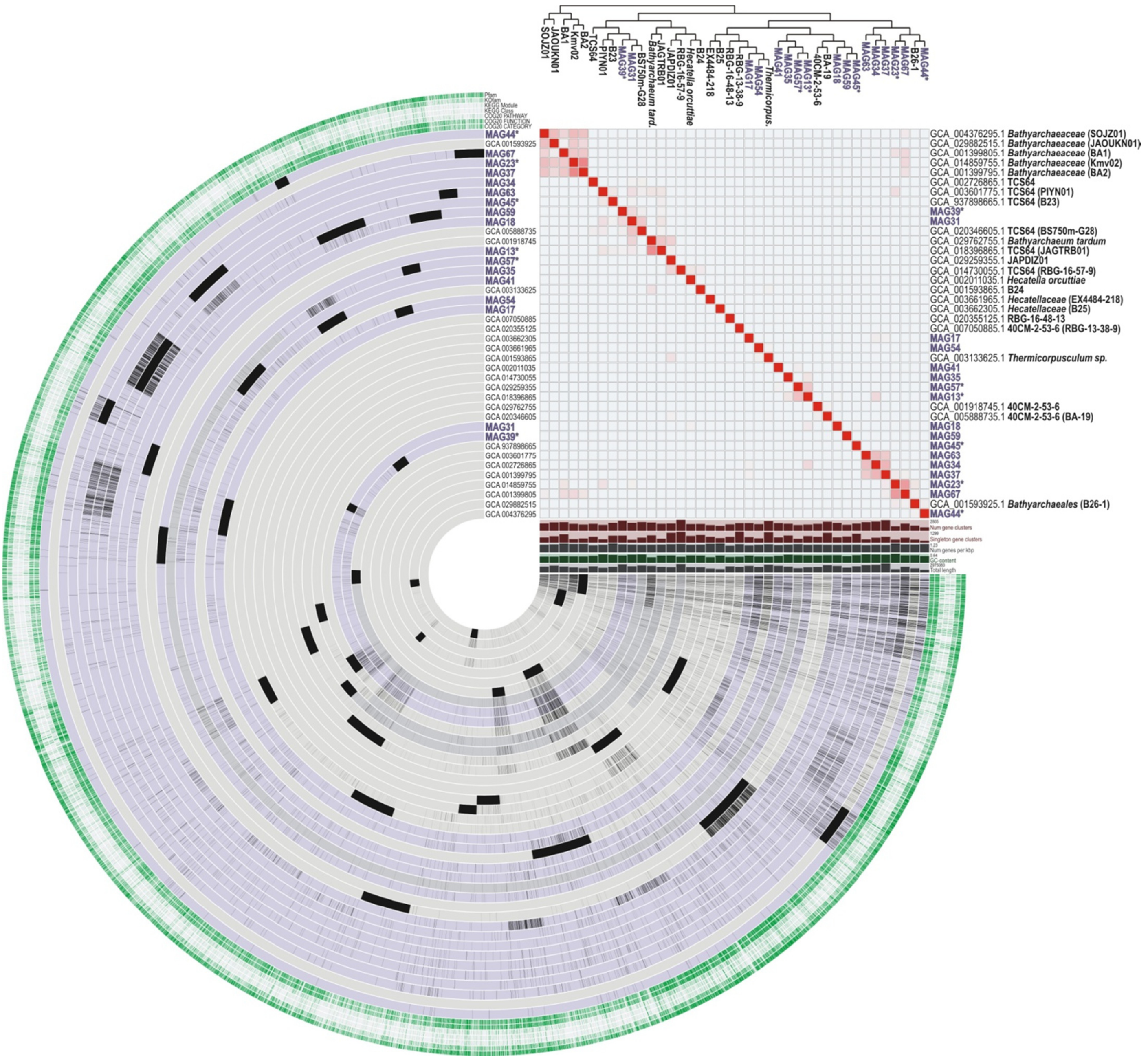

Supplementary Figure S10. Pangenomic analysis including the 17 partial metagenome-assembled genomes (MAGs) assigned to Bathyarchaeia in this study and 22 representative MAGs from the GTDB database [5]. Alignments and data visualization were performed in Anvi'o [12].

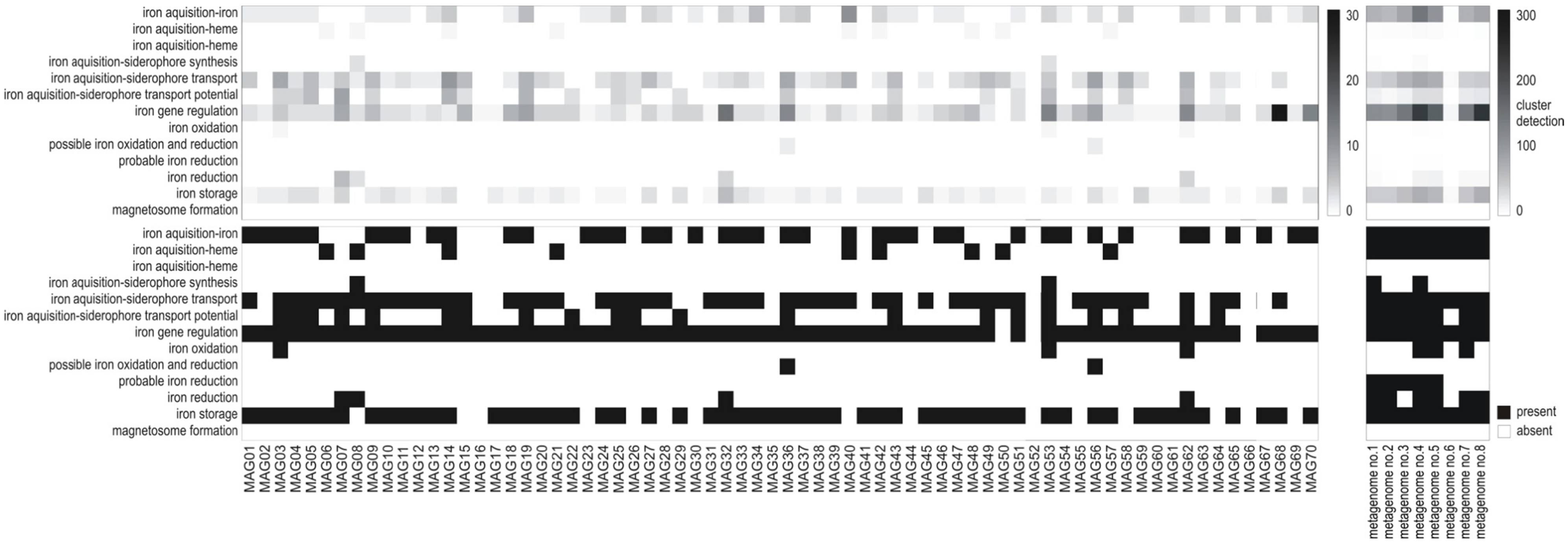

Supplementary Figure S11. Heatmaps of metabolic potential associated with iron processes based on the FeGenie pipeline [13].

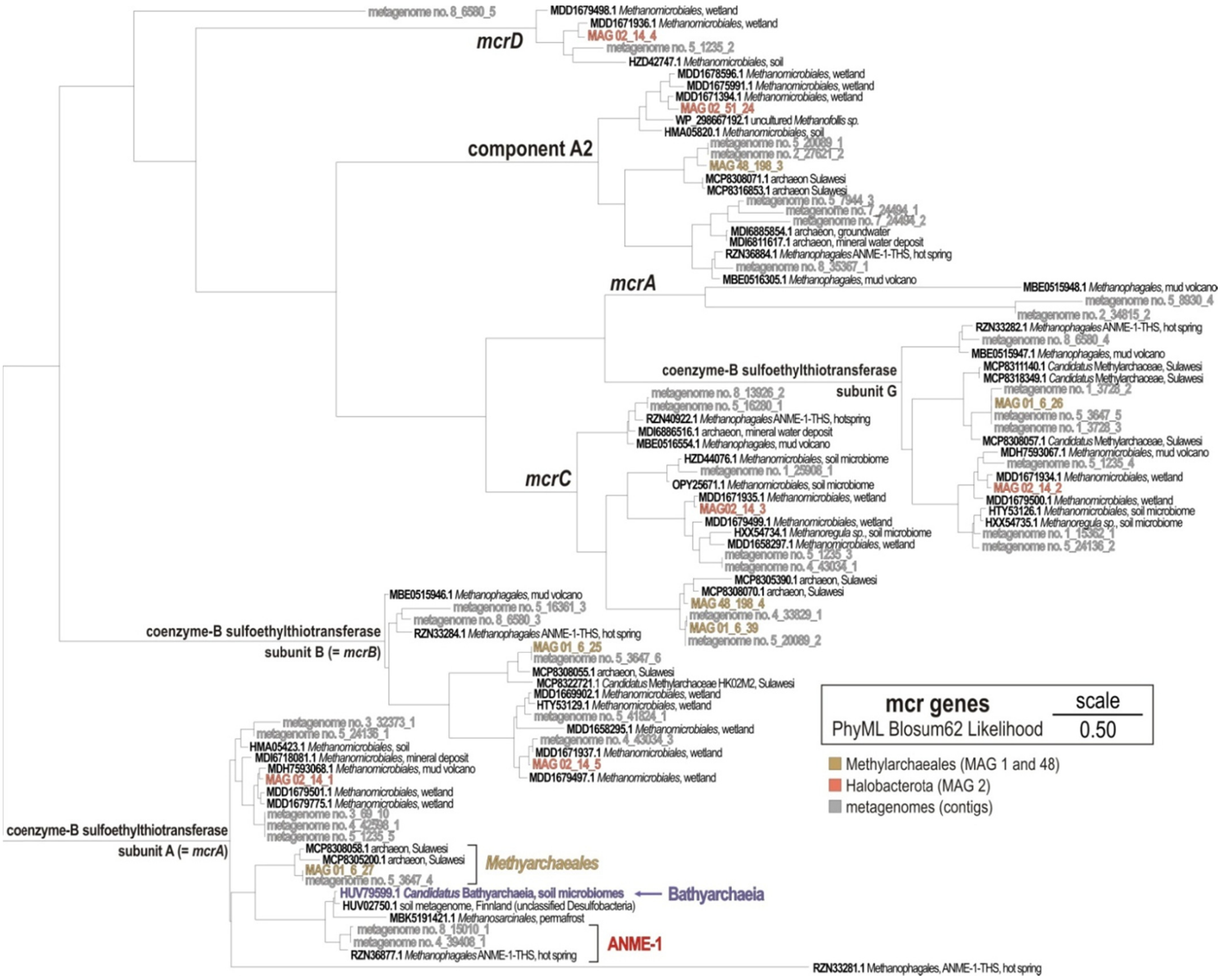

Supplementary Figure S12. Phylogenetic tree of methyl-coenzyme M reductase proteins. PhyML Blosum62 phylogenetic tree of MUSCLE-aligned regions for extracted Open Reading Frames (ORFs) encoding proteins of the different subunits of the methyl-coenzyme M reductase (*mcr*) and their closest hits to the NCBI database. None of the extracted *mcr* proteins were assigned to Bathyarcheia. Protein sequences were aligned using MUSCLE [14] and the PhyML Blosum62 [15] phylogenetic tree calculated based on 100 replicates, using Seaview v. 5 [16]. Boldface types signify sequence accession numbers to the NCBI database.

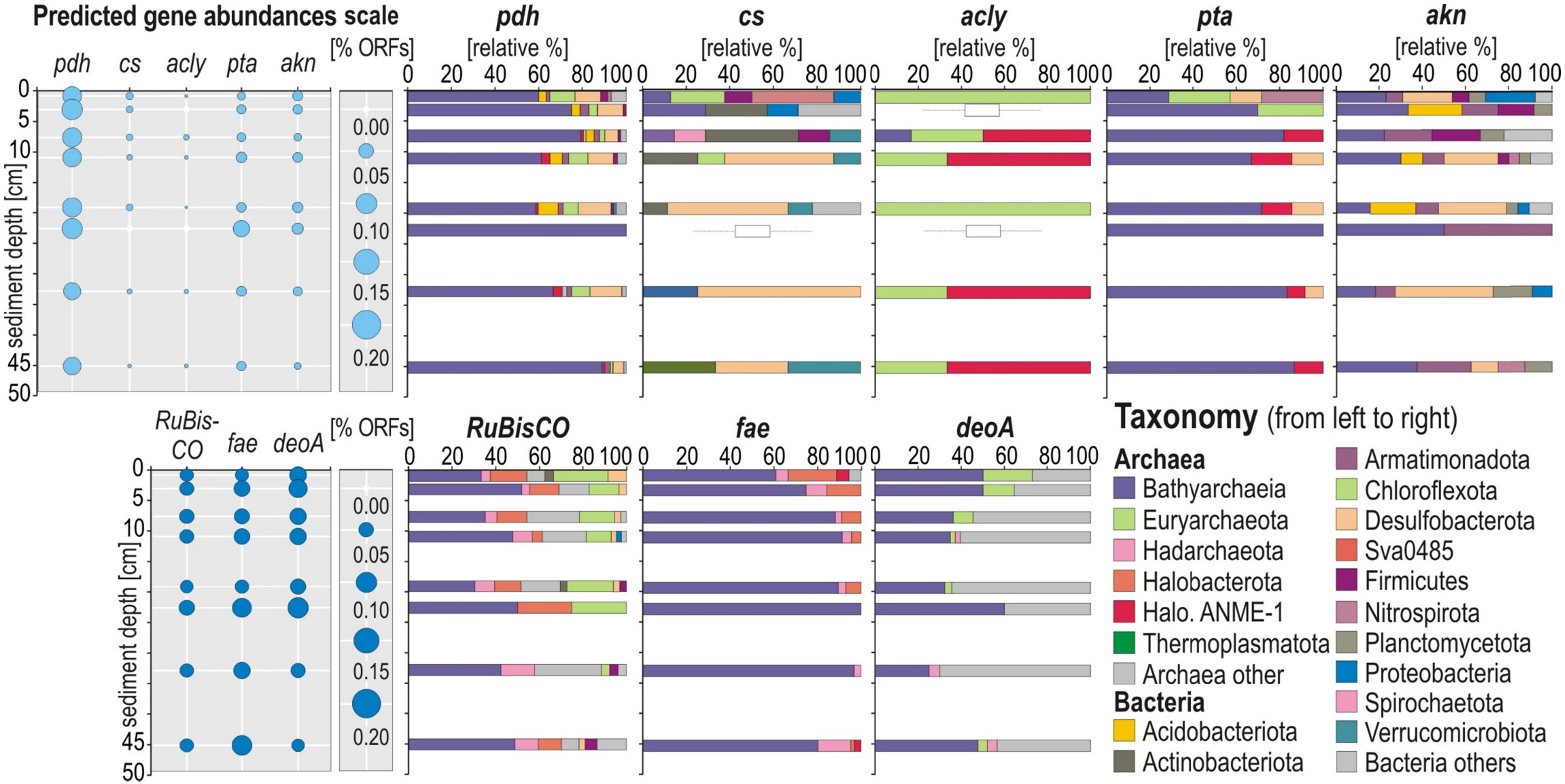

Supplementary Figure S13. Relative abundances of functional marker genes and their corresponding taxonomic assignments. Relative abundances (**left**) and taxonomic assignments (right) of open reading frames (ORFs) encoding metabolic steps involved in: (**1st row**) the transition from fermentative glycolysis (*pdh*) to TCA (*cs*) and rTCA (*acly*) cycle and substrate level phosphorylation of acetate (*pta*, *akn*); (**2nd row**) in the *RuBisCo*-mediated pathway in Archaea and its specific carbon substrates (*RuBisCO*, *fae*, *deoA*). All gene abbreviations are listed in Supplementary Table S1.

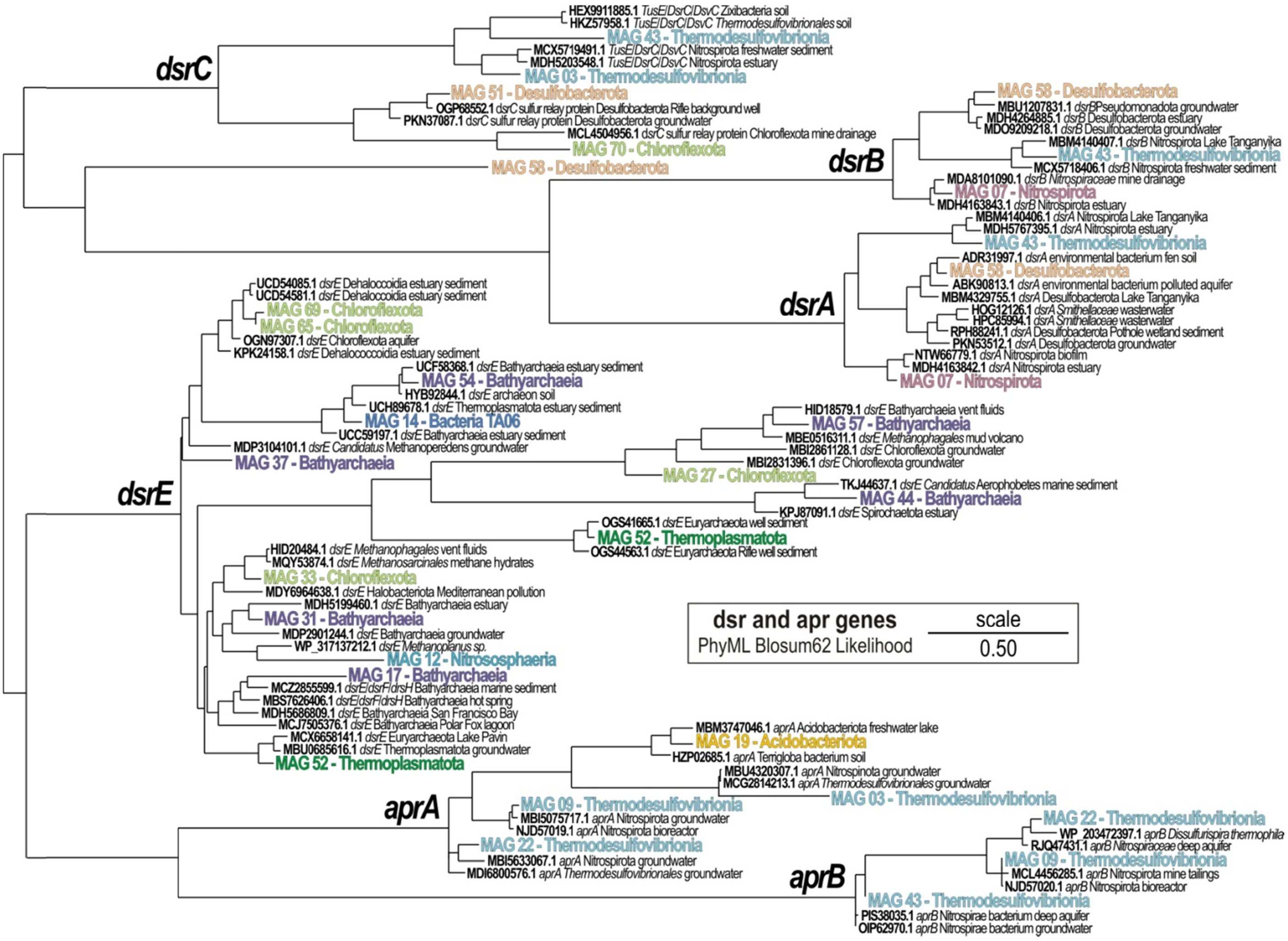

Supplementary Figure S14. Phylogenetic tree of dissimilatory sulfite reductase and adenylylsulfate reductase proteins. PhyML Blosum62 phylogenetic tree of MUSCLE-aligned regions for extracted Open Reading Frames (ORFs) encoding proteins of the different subunits of the dissimilatory sulfite reductase (*dsr*) and adenylylsulfate reductase (*apr*) and their closest hits to the NCBI database. The presence of the *dsrAB* and *aprAB* subunits confirms the MAGs assigned to the phyla Acidobacteriota, Nitrospirota and Desulfobacterota as potential sulfate-reducing bacteria (SRB). MAGs of Chloroflexota only include the subunit *dsrC* and *dsrE*, which are relevant to cellular sulfur reduction and assimilation [17]. Protein sequences were aligned using MUSCLE [14] and the PhyML Blosum62 [14] phylogenetic tree calculated based on 100 replicates, using Seaview v. 5 [16]. Boldface types signify sequence accession numbers to the NCBI database.

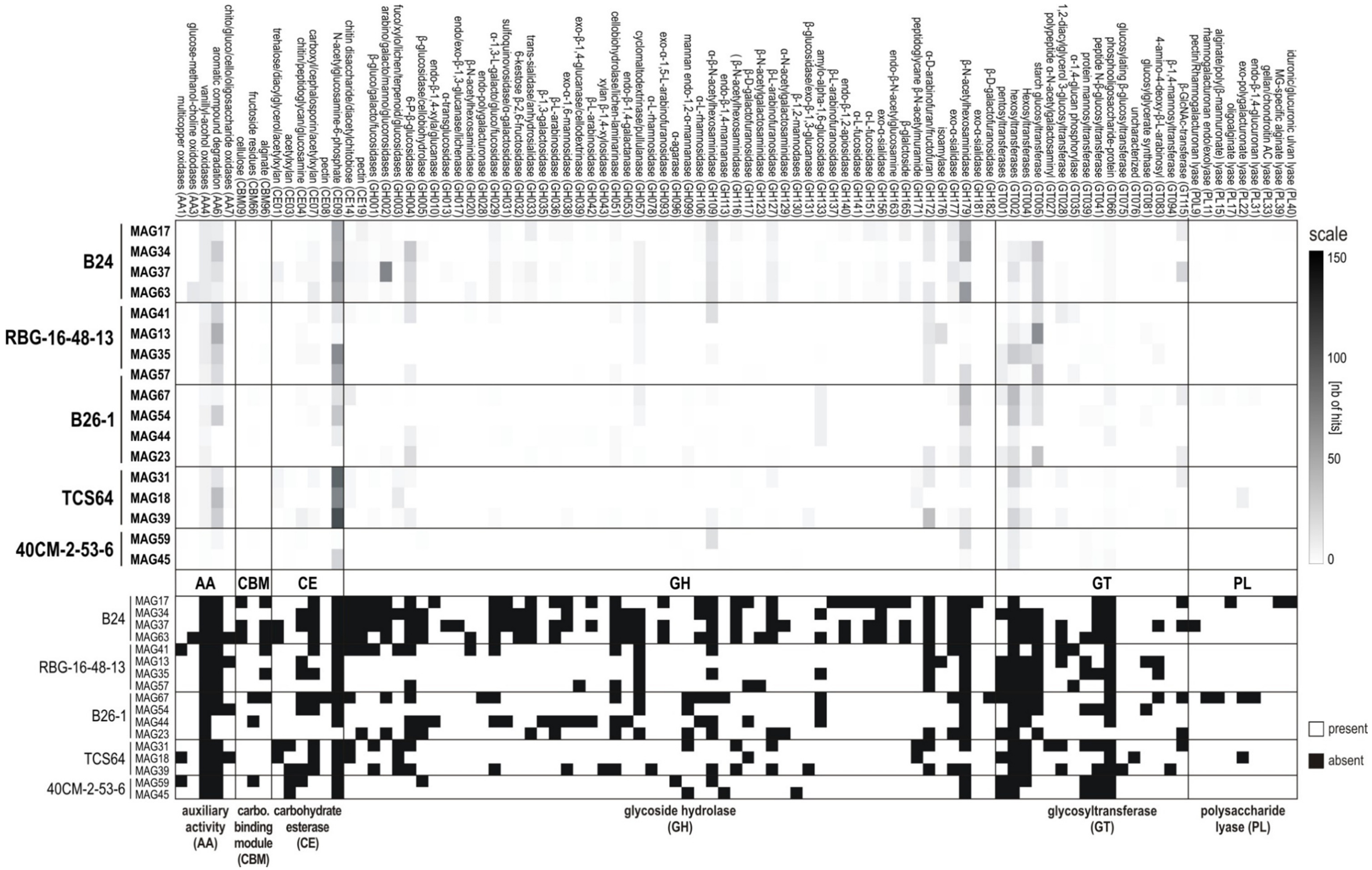

Supplementary Figure S15. Heatmap of the carbohydrates and carbohydrate-active enzymes (CAZymes) analysis performed on the 17 metagenome-assembled genomes assigned to Bathyarchaeia against the CAZy database [18] integrated in anvi'o [12].

Supplementary Tables

- **Supplementary Table S1.** List of enzymes and gene abbreviations from Open Reading Frames (ORFs).
- **Supplementary Table S2.** Statistics of *de novo* assembly for 8 separate metagenomic libraries and their co-assembly.
- **Supplementary Table S3.** Statistics of *de novo* co-assembly for archaeal metagenome-assembled genomes.
- **Supplementary Table S4.** Statistics of *de novo* co-assembly for bacterial metagenome-assembled genomes.

Supplementary Table S1. List of gene abbreviations extracted from Open Reading Frames (ORFs)

Processes and pathways	Enzymes	Gene abbreviations
dissimilatory sulfur metabolism	sulfate adenylyltransferase	*sat*
	adenylylsulfate reductase	*apr*
	dissimilatory sulfate reductase	*dsr*
	anaerobic sulfite reductase	*asr*
	thiosulfate/polysulfide reductase	*phs*/*psr*
	sulfhydrogenase	*hyd*
TCA cycle (1st step)	pyruvate dehydrogenase (glycolysis to TCA cycle)	*pdh*
	citrate synthase (1st step)	*cs*
rTCA cycle (1st step)	ATP citrate lyase (1st step)	*acly*
hydrogen production, proton pump	(non-) reducing F420 coenzyme hydrogenase	*frh*
	methyl-viologen hydrogenase	*mvh*
	heterodisulfide reductase	*hdr*
Wood-Ljungdahl pathway (carbonyl-branch) and acetate assimilation	formate dehydrogenase	*fdh*
	formate-tetrahydrofolate synthetase/ligase	*fts*
	5,10-methylenetetrahydrofolate cyclohydrolase/dehydrogenase	*mthfc/mthfd*
	5,10-methylenetetrahydrofolate reductase	*mthfr*
	methyl-tetrahydrofolate: corrinoid methyltransferase	*mthmt*
	phosphotransacetylase	*pta*
	acetate kinase	*akn*
	acetyl-coenzyme A synthetase	*acs*
	acyl-coenzyme A synthetase/ligase short chain (= acetate-CoA ligase)	*acss2*
Wood-Ljungdahl pathway (methyl-branch)	formylmethanofuran dehydrogenase	*fmd*
	formylmethanofuran: tetrahydromethanopterin formyltransferase	*ftr*
	5,10-methenyltetrahydromethanopterin cyclohydrolase	*mch*
	F420-dependent 5,10-methylene-tetrahydromethanopterin dehydrogenase	*mtd*
	5,10-methylene-tetrahydromethanopterin reductase	*mer*
	methanol coenzyme M methyltransferase	*mta*
	methylamine-specific coenzyme M methyltransferase	*mtb*
	methylthiol coenzyme M methytransferase	*mts*
	tetrahydromethanopterin-S-methyltransferase	*mtr*
	methyl-coenzyme M reductase	*mcr*
Wood-Ljungdahl pathway (both branches)	carbon monoxide dehydrogenase	*codh*
	acetyl-coenzyme A decarbonylase/synthase	*cdha*
electron transfer chain oxidoreductase	thioredoxin	*trx*
	rubredoxin	*rbx*
	glutaredoxin	*grx*
	aldehyde-ferredoxin (i.e. glycolysis)	*ald-fdx*
	2-oxoacid-ferredoxin (i.e. TCA cycle)	*2-oxo-fdx*
	pyruvate-ferredoxin (i.e. W-L pathway)	*py-fdx*
	sulfide dehydrogenase (aka bifurcating ferredoxin: NADP)	*sud* (*nfn*)
electron bifurcation complex	Energy-converting hydrogenase (membrane-bound, ferredoxin)	*Ech*
	Ion-translocating oxidoreductase Rnf complex	*Rnf*
	NADH-quinone oxidoreductase Nuo complex	*Nuo*
RuBiSCO pathway (reductive hexulose-phosphate) (AMP metabolism)	ribulose-1,5-diphosphate carboxylase-oxygenase	*RuBisCO*
	formaldehyde-activating enzyme	*fae*
	thymidine phosphorylase / AMP phosphorylase	*deoA*

Supplementary Table S2. Statistics of *de novo* assembly for 8 separate metagenomes and their co-assembly.

Sample depths	Total bases	Total reads	Assembly lengths [Mbps]	Assembled reads	Assembled reads [%]	Contig lengths [bps]	No. of contigs	No. of ORFs	No. of predicted genes	No. of bins	MAGs
0-1 cmblf	14,076,127,268	97,541,290	86.34	8,906,694	9.13	86,336,862	33,423	105,569	64,306	11	**43**
2-4 cmblf	11,993,571,227	83,660,094	96.42	10,469,768	12.51	96,418,278	36,674	116,557	68,928	11	
6-8 cmblf	12,253,873,851	84,399,842	117.86	13,918,851	16.49	117,864,272	40,459	141,305	83,161	15	
10-12 cmblf	13,828,497,284	95,871,790	154.50	19,314,120	20.15	154,501,980	53,586	184,808	107,570	19	
14-16 cmblf	12,414,592,012	85,740,172	126.01	15,184,396	17.71	126,004,992	42,355	149,292	88,901	18	
20-25 cmblf	3,653,333,261	24,739,826	10.37	1,126,404	4.55	10,373,324	3,853	13,422	7,943	1	
30-35 cmblf	8,570,735,106	59,279,164	93.80	13,039,484	22.00	93,798,608	32,274	113,899	67,049	15	
40-45 cmblf	10,543,055,517	72,911,328	130.83	17,169,983	23.55	130,830,790	43,551	159,542	97,419	16	
Average	***10,916,723,191***	***75,517,938***	***102***	***12,391,213***	***15.76***	***102,016,138***	***35,772***	***123,049***	***73,160***	***13***	
**Total**	87,333,785,526	604,143,506	-	99,129,700	-	-	286,175	-	616,552	-	
**Coassembly**	87,303,187,570	603,920,334	1,149.48	203,560,090	33.71	1,149,493,251	499,833	1,145,704	1,347,940	91	**70**
**70 MAGs**	-	-	151.83	34,100,356	16.75	151,484,619	26,144	93,247	65,693	-	-

Supplementary Table S3. Statistics of *de novo* co-assembly for archaeal metagenome-assembled genomes.

Archaea	Completeness	Contamination	predicted ORFs	GTDB Taxonomy
MAG46	67.10	4.00	638	Hadarchaeota B88-G9
MAG21	74.02	4.58	883	Halobacteriota Methanocellia Methanocellales
MAG02	95.62	0.65	1156	Halobacteriota Methanomicrobia Methanomicrobiales JACTUA01
MAG50	63.35	0.00	670	Halobacteriota Methanomicrobia Methanomicrobiales JACTUA01
MAG42	87.37	6.89	987	Methanobacteriota_B Thermococci Methanofastidiosales SZ-28-30
MAG16	69.39	0.00	348	Nanoarchaeota Nanoarchaeia Woesearchaeales ARS49
MAG38	71.09	3.20	782	Thermoplasmatota Thermoplasmata RBG-16-68-12
MAG06	97.47	1.60	1463	Thermoplasmatota Thermoplasmata UBA10834 UBA10834
MAG52	64.45	2.34	993	Thermoplasmatota Thermoplasmata UBA10834 UBA10834
MAG59	90.65	4.67	851	Thermoproteota Bathyarchaeia 40CM-2-53-6
MAG45	80.34	6.80	1061	Thermoproteota Bathyarchaeia 40CM-2-53-6 FEN-987
MAG17	97.09	2.91	1053	Thermoproteota Bathyarchaeia B24 JAGTQN01
MAG34	97.09	5.34	1138	Thermoproteota Bathyarchaeia B24 JAGTQN01
MAG37	94.17	4.37	1249	Thermoproteota Bathyarchaeia B24 JAGTQN01
MAG63	92.23	5.83	1016	Thermoproteota Bathyarchaeia B24 JAGTQN01
MAG23	58.65	0.93	850	Thermoproteota Bathyarchaeia B26-1
MAG44	61.08	2.43	700	Thermoproteota Bathyarchaeia B26-1 B26-1
MAG67	96.26	6.85	1228	Thermoproteota Bathyarchaeia B26-1 BA1
MAG54	90.34	1.87	1337	Thermoproteota Bathyarchaeia B26-1 UBA233
MAG41	87.07	3.74	1074	Thermoproteota Bathyarchaeia RBG-16-48-13
MAG35	78.07	3.58	960	Thermoproteota Bathyarchaeia RBG-16-48-13
MAG13	82.77	7.77	825	Thermoproteota Bathyarchaeia RBG-16-48-13
MAG57	63.50	7.48	820	Thermoproteota Bathyarchaeia RBG-16-48-13
MAG18	88.83	4.85	1147	Thermoproteota Bathyarchaeia TCS64 PIYN01
MAG31	90.19	1.87	1579	Thermoproteota Bathyarchaeia TCS64 TCS64
MAG39	81.38	9.57	1794	Thermoproteota Bathyarchaeia TCS64 TCS64
MAG61	85.93	4.85	885	Thermoproteota EX4484-205 EX4484-205 DTQO01 (= Brockarchaeota)
MAG66	72.82	0.00	186	Thermoproteota EX4484-205 EX4484-205 JAAOZO01 (= Brockarchaeota)
MAG60	70.79	2.80	443	Thermoproteota Methanomethylicia (= Candidatus Verstraetearchaeota)
MAG12	99.03	2.91	803	Thermoproteota Nitrososphaeria JACIWG01 JACIWG01 JACIWG01
MAG01	99.51	0.97	771	Thermoproteota Nitrososphaeria Nitrososphaerales JACAEJ01 JACAEJ01 (= Methylarchaeales)
MAG48	74.62	4.85	740	Thermoproteota Nitrososphaeria Nitrososphaerales JACAEJ01 JACAEJ01 (= Methylarchaeales)

Supplementary Table S4. Statistics of *de novo* co-assembly for bacterial metagenome-assembled genomes.

Bacteria	Completeness	Contamination	predicted ORFs	GTDB Taxonomy
MAG15	70.35	0.00	987	Acidobacteriota Acidobacteriae Acidoferrales UBA7541
MAG19	84.18	1.71	1454	Acidobacteriota Acidobacteriae UBA7540 UBA7540
MAG53	90.80	3.04	2038	Acidobacteriota Aminicenantia Aminicenantales Aminicenantaceae
MAG36	80.97	1.71	1842	Acidobacteriota Aminicenantia Aminicenantales Aminicenantaceae
MAG56	72.71	5.22	1963	Acidobacteriota Aminicenantia Aminicenantales RBG-16-66-30
MAG08	96.25	1.53	894	Actinobacteriota Coriobacteriia OPB41 D1FN1-002 D1FN1-002 D1FN1-002 sp005774595
MAG62	95.61	0.65	1732	Bacteroidota UBA10030 UBA10030 UBA10030 VGWB01
MAG24	60.12	0.00	1241	Chloroflexota Anaerolineae E26-bin7 E26-bin7 E26-bin7
MAG70	74.24	5.31	3003	Chloroflexota Anaerolineae Thermoflexales Fen-1058
MAG33	82.26	4.09	1220	Chloroflexota Anaerolineae UBA7937
MAG65	74.26	2.38	729	Chloroflexota Dehalococcoidia Dehalococcoidales E44-bin46 E44-bin46
MAG69	70.46	0.00	710	Chloroflexota Dehalococcoidia Dehalococcoidales E44-bin46 E44-bin46
MAG27	82.89	3.96	1398	Chloroflexota Dehalococcoidia Dehalococcoidales RBG-16-60-22
MAG30	70.84	0.00	1099	Chloroflexota Dehalococcoidia Dehalococcoidales RBG-16-60-22 E44-bin89
MAG20	70.63	4.57	1543	Chloroflexota Dehalococcoidia Dehalococcoidales UBA2162
MAG11	93.73	1.49	1447	Chloroflexota Dehalococcoidia E44-bin15 E44-bin15 Kmv38
MAG55	71.55	3.83	1173	Chloroflexota Dehalococcoidia RBG-13-53-26 RBG-13-53-26
MAG28	63.70	0.00	849	Chloroflexota Dehalococcoidia RBG-13-53-26 RBG-13-53-26
MAG47	73.77	1.73	1887	Chloroflexota Dehalococcoidia SZUA-161
MAG05	98.06	0.65	1776	Desulfobacterota Desulfomonilia UBA1062 UBA1062 MWEI01
MAG25	77.72	1.93	1686	Desulfobacterota Desulfomonilia UBA1062 UBA1062 MWEI01
MAG04	94.19	0.00	1772	Desulfobacterota Desulfuromonadia Desulfuromonadales BM103 VAUL01
MAG58	79.65	3.23	2729	Desulfobacterota SM23-61 SM23-61 SM23-61 JACRCA01
MAG51	87.90	4.25	2455	Desulfobacterota Syntrophia Syntrophales Smithellaceae Smithella
MAG49	81.86	1.94	1363	Desulfobacterota WTBG01
MAG29	84.87	4.44	1407	Methylomirabilota Methylomirabilia DTKO01 DTKO01
MAG40	58.36	0.11	1799	Methylomirabilota Methylomirabilia Rokubacteriales CSP1-6
MAG03	96.82	0.91	1673	Nitrospirota Thermodesulfovibrionia Thermodesulfovibrionales SM23-35 JACAEY01
MAG43	84.92	9.57	1753	Nitrospirota Thermodesulfovibrionia Thermodesulfovibrionales SM23-35 JACAEY01
MAG22	83.02	1.82	1821	Nitrospirota Thermodesulfovibrionia Thermodesulfovibrionales UBA6898
MAG09	97.27	0.91	1819	Nitrospirota Thermodesulfovibrionia Thermodesulfovibrionales UBA6898 JACRHE01
MAG64	64.88	0.91	1425	Nitrospirota Thermodesulfovibrionia Thermodesulfovibrionales UBA6898 PALSA-1316
MAG07	96.59	0.91	1931	Nitrospirota UBA9217 UBA9217 UBA9217
MAG32	78.46	2.41	3094	Planctomycetota Phycisphaerae Sedimentisphaerales SG8-4
MAG10	77.84	2.27	1287	Planctomycetota Phycisphaerae Sedimentisphaerales SG8-4 CAIYOL01
MAG68	95.45	8.71	3622	Planctomycetota Phycisphaerae Sedimentisphaerales SG8-4 JAFNGF01
MAG14	95.10	2.20	1051	TA06 DG-26 E44-bin18
MAG26	64.22	0.00	1145	Zixibacteria MSB-5A5 MSB-5A5 RBG-16-43-9 RBG-16-43-9

Supplementary Methods

Eight metagenomes were generated from DNA extracts (at 0-1, 2-4, 6-8, 10-12, 14-16, 20-25, 30-35, and 40-45 cmblf), using the Nextera XT DNA Library Preparation kit (Illumina). Sequencing was performed on a NovaSeq 6000 Illumina platform at CeGaT GmbH (Tübingen, Germany), aiming for 50 million read pairs (2 × 150 bps) for each sample. Library demultiplexing was performed with bcl2fastq2 v. 2.20. Adapters were trimmed with Skewer v. 0.2.2 [19], and FASTQ files quality-checked using FastQC v. 0.11.5. Quality-controlled reads were mapped to the SILVA 16S rRNA SSU database release 138 [20], using Bowtie2 [21]. Metagenomic reads were further processed for quality control, *de novo* assembly of contigs, gene annotation, binning into MAGs and taxonomic annotation, using ATLAS v. 2.1.0 [22]. Metagenomes were initially processed separately, then combined to improve the completeness of MAGs. The integrated ATLAS pipeline uses quality-controlled reads from BBTools [23] to successively execute metaSPAdes v. 3.11.1 [24] for contig assembly, Prodigal v. 2.6.3 [25] for Open Reading Frames (ORFs) extraction with eggNOG-mapper v. 2.1. [26] for functional annotation, MetaBAT v. 2.1.5 [27] with MaxBin v. 2.2.7 [28] and DAS Tool v. 1.1.6 [29] for binning into MAGs, and CheckM v.1.1.10 [30] to determine the level of MAG completeness and contamination. Taxonomic assignments of the MAGs were performed against the GTDB Genome Taxonomy Database v. 2.1.1 [5]. In addition, we extracted 16S rRNA gene sequences that could be assembled in our MAGs and plotted them in a phylogenetic tree (Supplementary Fig. S8), as described above.

Taxonomic identifications integrated with functional annotations were performed on all ORFs extracted from contigs assembled from separate metagenomes, using DIAMOND protein aligner v. 0.9.24 [31]. BLASTp searches were run against an aggregated genome database of 37.8 million predicted proteins, including the SEED [32] and NCBI RefSeq databases, and taxonomy of the best hit assigned to the corresponding ORF, as published [33, 34]. We were thus able to attribute the microbial provenance of all ORFs to high taxonomic levels [35] and exert comparison with the genetic content of MAGs assembled from combined metagenomes.

Supplementary References

1. Pruesse E, Quast C, Knittel K, Fuchs BM, Ludwig W, Peplies J, et al. SILVA: A comprehensive online resource for quality checked and aligned ribosomal RNA sequence data compatible with ARB. *Nucleic Acids Res.* 2007; **35**: 7188–7196.
2. Quast C, Pruesse E, Yilmaz P, Gerken J, Schweer T, Yarza P, et al. The SILVA ribosomal RNA gene database project: improved data processing and web-based tools. *Nucleic Acids Res.* 2013; **41**: D590–D596.
3. Ludwig W, Strunk O, Westram R, Richter L, Meier H, Yadhukumar, et al. ARB: A software environment for sequence data. *Nucleic Acids Res.* 2004; **32**: 1363–1371.
4. Hammer O, Harper D, Ryan P. PAST: Paleontological statistics software package for education and data analysis. *Palaeont Electr.* 2001; **4**: 1–9.
5. Parks DH, Chuvochina M, Rinke C, Mussig AJ, Chaumeil P-A, Hugenholtz P. GTDB: An ongoing census of bacterial and archaeal diversity through a phylogenetically consistent, rank normalized and complete genome-based taxonomy. *Nucleic Acids Res.* 2022; **50**: D785–D794.
6. Kieser S, Brown J, Zdobnov EM, Trajkovski M, McCue LA. ATLAS: A Snakemake workflow for assembly, annotation, and genomic binning of metagenome sequence data. *BMC Bioinformatics* 2020; **21**: 257.
7. Graham E, Tully B. Building phylogenetic tree. *protocols.io* 2018; 9693:dx.doi.org/10.17504/protocols.io.mp5c5q6
8. Graham ED, Heidelberg JF, Tully BJ. Potential for primary productivity in a globally-distributed bacterial phototroph. *ISME J.* 2018; **12**: 1861–1866.
9. Letunic I, Bork P. Interactive Tree of Life (iTOL) v6: Recent updates to the phylogenetic tree display and annotation tool. *Nucleic Acids Res.* 2024; **in press**: gkae268.
10. Khomyakova MA, Merkel AY, Mamiy DD, Klyukina AA, Slobodkin AI. Phenotypic and genomic characterization of *Bathyarchaeum tardum* gen. nov., sp. nov., a cultivated representative of the archaeal class Bathyarchaeia. *Front Microbiol* 2023; **14**: 1214631.
11. Adam PS, Kolyfetis GE, Bornemann TLV, Vorgias CE, Probst AJ. Genomic remnants of ancestral methanogenesis and hydrogenotrophy in Archaea drive anaerobic carbon cycling. *Sci Adv.* 2022; **8**: eabm9651.
12. Eren AM, Esen ÖC, Quince C, Vineis JH, Morrison HG, Sogin ML, et al. Anvi'o: An advanced analysis and visualization platform for 'omics data. *PeerJ* 2015; **3**: e1319.
13. Garber AI, Nealson KH, Okamoto A, McAllister SM, Chan CS, Barco RA, et al. FeGenie: A comprehensive tool for the identification of iron genes and iron gene neighborhoods in genome and metagenome assemblies. *Front Microbiol* 2020; **11**: 37.

14. Edgar RC. MUSCLE: Multiple sequence alignment with high accuracy and high throughput. *Nucleic Acids Res.* 2004; **32**: 1792–1797.

15. Guindon S, Dufayard J-F, Lefort V, Anisimova M, Hordijk W, Gascuel O. New algorithms and methods to estimate Maximum-Likelihood Phylogenies: Assessing the performance of PhyML 3.0. *Syst Biol.* 2010; **59**: 307–321.

16. Gouy M, Guindon S, Gascuel O. SeaView version 4: A multiplatform graphical user interface for sequence alignment and phylogenetic tree building. *Mol Biol Evol.* 2010; **27**: 221–224.

17. Venceslau SS, Stockdreher Y, Dahl C, Pereira IAC. The "bacterial heterodisulfide" DsrC is a key protein in dissimilatory sulfur metabolism. *Biochim Biophys Acta* 2014; **1837**: 1148–1164.

18. Cantarel BL, Coutinho PM, Rancurel C, Bernard T, Lombard V, Henrissat B. The Carbohydrate-Active enZymes database (CAZy): An expert resource for glycogenomics. *Nucleic Acids Res.* 2009; **37**: D233–D238.

19. Jiang H, Lei R, Ding S-W, Zhu S. Skewer: a fast and accurate adapter trimmer for next-generation sequencing paired-end reads. *BMC Bioinformatics* 2014; **15**: 182.

20. Quast C, Pruesse E, Yilmaz P, Gerken J, Schweer T, Yarza P, et al. The SILVA ribosomal RNA gene database project: Improved data processing and web-based tools. *Nucleic Acids Res.* 2013; **41**: D590–D596.

21. Langmead B, Salzberg SL. Fast gapped-read alignment with Bowtie 2. *Nat Methods* 2012; **9**: 357–359.

22. Kieser S, Brown J, Zdobnov EM, Trajkovski M, McCue LA. ATLAS: a Snakemake workflow for assembly, annotation, and genomic binning of metagenome sequence data. *BMC Bioinformatics* 2020; **21**: 257.

23. Bushnell B. BBMap: A fast, accurate, splice-aware aligner. 2014; https://sourceforge.net/projects/bbmap/

24. Nurk S, Meleshko D, Korobeynikov A, Pevzner PA. metaSPAdes: a new versatile metagenomic assembler. *Genome Res.* 2017; **27**: 824–834.

25. Hyatt D, Chen G-L, LoCascio PF, Land ML, Larimer FW, Hauser LJ. Prodigal: Prokaryotic gene recognition and translation initiation site identification. *BMC Bioinformatics* 2010; **11**: 119.

26. Cantalapiedra CP, Hernández-Plaza A, Letunic I, Bork P, Huerta-Cepas J. eggNOG-mapper v2: Functional annotation, orthology assignments, and domain prediction at the metagenomic scale. *Mol Biol Evol.* 2021; **38**: 5825–5829.

27. Kang DD, Li F, Kirton E, Thomas A, Egan R, An H, et al. MetaBAT 2: An adaptive binning algorithm for robust and efficient genome reconstruction from metagenome assemblies. *PeerJ* 2019; **7**: e7359.

28. Wu Y-W, Simmons BA, Singer SW. MaxBin 2.0: An automated binning algorithm to recover genomes from multiple metagenomic datasets. *Bioinformatics* 2016; **32**: 605–607.

29. Sieber CMK, Probst AJ, Sharrar A, Thomas BC, Hess M, Tringe SG, et al. Recovery of genomes from metagenomes via a dereplication, aggregation and scoring strategy. *Nat Microbiol.* 2018; **3**: 836–843.

30. Parks DH, Imelfort M, Skennerton CT, Hugenholtz P, Tyson GW. CheckM: assessing the quality of microbial genomes recovered from isolates, single cells, and metagenomes. *Genome Res.* 2015; **25**: 1043–1055.

31. Buchfink B, Xie C, Huson DH. Fast and sensitive protein alignment using DIAMOND. *Nat Methods* 2015; **12**: 59–60.

32. Overbeek R, Begley T, Butler RM, Choudhuri JV, Chuang H-Y, Cohoon M, et al. The subsystems approach to genome annotation and its use in the project to annotate 1000 genomes. *Nucleic Acids Res.* 2005; **33**: 5691–5702.

33. Orsi WD, Richards TA, Francis WR. Predicted microbial secretomes and their target substrates in marine sediment. *Nature Microbiology* 2018; **3**: 32–37.

34. Orsi WD, Vuillemin A, Coskun ÖK, Rodriguez P, Oertel Y, Niggemann J, et al. Carbon assimilating fungi from surface ocean to subseafloor revealed by coupled phylogenetic and stable isotope analysis. *ISME J.* 2022; **16**: 1245–1261.

35. Orsi WD, Vuillemin A, Rodriguez P, Coskun ÖK, Gomez-Saez GV, Lavik G, et al. Metabolic activity analyses demonstrate that Lokiarchaeon exhibits homoacetogenesis in sulfidic marine sediments. *Nat Microbiol.* 2020; **5**: 248–255.
